# Effects of shape and solute-solvent compatibility on the efficacy of chirality transfer: Nanoshapes in nematics

**DOI:** 10.1126/sciadv.abl4385

**Published:** 2022-01-26

**Authors:** Ahlam Nemati, Lara Querciagrossa, Corinne Callison, Sasan Shadpour, Diana P. Nunes Gonçalves, Taizo Mori, Ximin Cui, Ruoqi Ai, Jianfang Wang, Claudio Zannoni, Torsten Hegmann

**Affiliations:** 1 Materials Science Graduate Program, Kent State University, Kent, OH, USA.; 2 Advanced Materials and Liquid Crystal Institute, Kent State University, Kent, OH, USA.; 3 Dipartimento di Chimica Industriale and INSTM, Università di Bologna, Bologna, Italy.; 4 Institute for Solid State Physics, The University of Tokyo, Tokyo, Japan.; 5 Department of Physics, The Chinese University of Hong Kong, Shatin, Hong Kong SAR, China.; 6 Department of Chemistry and Biochemistry, Kent State University, Kent, OH, USA.; 7 Brain Health Research Institute, Kent State University, Kent, OH, USA.

## Abstract

Chirality, as a concept, is well understood at most length scales. However, quantitative models predicting the efficacy of the transmission of chirality across length scales are lacking. We propose here a modus operandi for a chiral nanoshape solute in an achiral nematic liquid crystal host showing that that chirality transfer may be understood by unusually simple geometric considerations. This mechanism is based on the product of a pseudoscalar chirality indicator and of a geometric shape compatibility factor based on the two-dimensional isoperimetric quotients for each nanoshape solute. The model is tested on an experimental set of precisely engineered gold nanoshapes. These libraries of calculated and in-parallel acquired experimental data among related nanoshapes pave the way for predictive calculations of chirality transfer in nanoscale, macromolecular, and biological systems, from designing chiral discriminators and enantioselective catalysts to developing chiral metamaterials and understanding nature’s innate ability to transfer homochirality across length scales.

## Teaser

Chirality transfer efficacy can be a priori predicted by a pseudoscalar chirality index corrected by a shape compatibility factor.

## INTRODUCTION

Chirality—the absence of mirror symmetry—is a fundamental concept involved in the creation (*1*–*5*), assembly (*6*, *7*), and specificity of biological (*8*), chemical (*9*), mechanical (*10*), and optical properties (*11*–*14*) of materials and is intricately woven into the fabric of the origin of life itself (*15*, *16*). Any physical object, phenomenon (*17*), or physical force (photons and electrons) (*18*) that cannot be precisely mapped upon its specular image by any kind of rotation or translations is said to be chiral. Following this classic definition by Kelvin (*19*, *20*), the presence or absence of chirality is reasonably well understood at length scales ranging from subatomic particles, atoms, and molecules (*21*) to supramolecular assemblies (*22*), nanoscale particles (*23*), all the way to macroscopic physical objects in flora and fauna (*24*). In addition to the binary nature of the concept, a quantitative metric of chirality can be established when chiral objects interact with a suitable external agent, e.g., when molecules interact with electromagnetic fields (*25*). Enantiomers, for example, will rotate the plane of linearly polarized light by the same magnitude in opposite directions and give rise to mirror image electronic circular dichroism (CD) spectra (*26*–*28*). Moreover, even the most diminutive changes to chemical structures, such as isotopic substitutions breaking an existing mirror symmetry, can lead to measurable differences in specific rotation or optical rotatory power that permit quantifying chirality (*29*, *30*). For mesogenic molecules, these minor changes can even alter a liquid crystal (LC) phase from an achiral nematic to a chiral nematic (or cholesteric) phase (*31*). Various experimental techniques and systems give comparable numerical values. These include the most frequently used quantitative experimental measures of chirality such as the anisotropic or Kuhn’s dissymmetry factors (*32*) for signals originating from electronic excitation (absorption) (*33*) or emission (luminescence) processes (*34*) [*g*_abs_ = 2(ε_L_ − ε_R_)/(ε_L_ + ε_R_) and *g*_lum_ = 2(*I*_L_ − *I*_R_)/(*I*_L_ + *I*_R_), with ε and *I* referring, respectively, to the molar absorption coefficient and the emission intensity of left- and right-handed circular polarized light]. Bridging experiment and theory leads to the quantifiable measures of the chirality parameter, κ = (*n*_+_ − *n*_−_)/2, in which *n*_+_ and *n*_−_ are the effective refractive indices of left- and right-handed circularly polarized waves, and the chiral polarization tensor α_c_ (*35*). Independent from experimental data, there are also several purely geometrical measures of chirality. One is based on the distance between a given chiral structure and its closest achiral motif, termed the continuous chirality measure (*36*); another one, the Hausdorff distance, measures the distances between sets of points representing two specular images (termed the Hausdorff chirality measure or HCM) (*37*, *38*). Contrasting this, there is an absolute (as opposed to being relative to a reference) pseudoscalar chirality indicator derived from a molecule’s or object’s geometry, termed the average chirality index, $\langle G_{\text{oa}}^{a}\rangle$(*39*–*41*). Apart from $\langle G_{\text{oa}}^{a}\rangle$, which provided decidedly meaningful insights on a thus far limited sample of chiral structures, including gold nanoshapes capped with a chiral ligand shell (*40*), none of these experimental, theoretical, or geometric chirality measures are capable of quantifying chirality transfer or amplification through space or across length scales. Aside from field-mediated chirality transfer from a chiral molecule to another achiral molecule through space, chirality transfer, for example, facilitated by a plasmonic nanoparticle in an electromagnetic field (*42*), arises from molecule-molecule or molecule-surface interactions through chemical bonds, intermolecular forces, or self-assembly and includes descriptive concepts such as sergeants-and-soldiers (*43*), self-sorting, or majority-rules effects (*44*).

Early Monte Carlo simulations of helical induction from a chiral surface to a nematic matrix (*45*) have indicated that the nematic molecules twist in the immediate proximity of the surface, while the LC orientational correlations propagate the effect. However, even in this case, the origin of the surface-LC transfer treated as parameters remains unknown. Here, we attempt to establish a minimalistic model of the effect that underpins the efficacy of chirality transfer to a surrounding medium that we believe is inherently connected to the shape of a carrier of chiral information (i.e., the solute, here in the form of a nanoscale object covered with chiral molecules). We assume in this model that there is a combination of the solute’s intrinsic chirality and its ability to twist the nematic host molecules (*N*) to transfer chirality across length scales. For a certain chiral solute *X*, we will thus assume that the helical twisting power (b), which as we shall see later, can be experimentally measured, can be written as

$$\beta \propto G^{X}S^{\mathrm{XN}}$$ (1)

in which *G^X^* is some suitable absolute chirality indicator for the solute or, with respect to the current experimental system, a nanoparticle, and *S^XN^* is a compatibility factor representing a solute-solvent transmission coefficient of the chiral solute to the achiral mesogen *N*. Here, we use for the first term the chirality indicator $|G_{\text{oa},\text{max}}^{a}|$, which can be obtained from atomistic structures of the chiral molecules on a nanoshape surface (*40*). $|G_{\text{oa},\text{max}}^{a}|$ is the absolute maximum value of the average $\langle G_{\text{oa}}^{a}\rangle$values obtained for the various ligand orientations (angles) with respect to a main axis as described in section S7.2. As for the second term, *S^XN^*, we assume it is based on a shape complementarity model quantifying shape resemblance or, in contrast, shape incongruity between the nematic LC (N-LC) molecule serving as reporter medium and a chiral molecule surface-modified nanoshape. A useful concept is provided by dimensionless isoperimetric ratios, IPR_nD_; scale-independent, shape-related metrics in *n* dimensions, commonly used to find assemblies of shapes in two-dimensional (2D) or 3D (i.e., *n* = 2, 3) that minimize the contact surface for a given amount of material (*46*). For instance, a classical example in 2D, in which the isoperimetric ratio is defined as IPR_2D_ = ℓ^2^/*A*, with ℓ being the length of the perimeter and *A* the surface area of a 2D shape, is the honeycomb arrangement that fills space with the best surface-to-area ratio. The similar problem in 3D, where the isoperimetric ratio is defined as IPR_3D_ = *A*^3^/*V*^2^, with *V* as the volume of the 3D object [e.g., used in Kelvin’s conjecture (*46*)], requires attempts to fill space with shapes, reducing the volume left to gaps and is much more complex, although well studied for systems such as foams (*47*).

As an ansatz, we assume here that the *S^XN^* can be related to the inverse difference between the 2D isoperimetric ratios of solute and solvent, $1/|\Delta \;{\text{IPR}}_{2D}^{\mathrm{XN}}|=1/(|{\text{IPR}}_{2D}^{X}-{\text{IPR}}_{2D}^{N}|)$. The degree of correlation between *S^XN^* and β will thus provide direct insights into the role of shape complementarity in chirality transfer. *S^XN^* is a scalar quantity to the degree that the orientation of one particle with respect to another is nonzero and scales invariantly under all spatial transformations, even in the achiral case. We have put the model to the test using a highly tunable experimental system to collect data ascertaining the chirality transfer efficacy of chiral molecule-capped plasmonic nanoshapes in a reporter medium formed by an achiral N-LC phase (*40*), as we shall now describe in detail.

## RESULTS AND DISCUSSION

### Chiral ligand–capped gold nanoshapes in an N-LC reporter medium

Engineering dynamic structures involving chiral nanoshapes with specific geometry, aspect ratio, and overall dimensions offers one possible strategy (*10*) to understand induction, transmission, and amplification of chirality through space. Another strategy may use a reporter medium that allows one to quantify the transmission efficacy of chiral information from one object to another (*48*). To acquire this knowledge, the induced chiral N-LC (N*-LC) phase, as a responsive and birefringent medium, is used here because the induced helical arrangement of the spatial orientation of the constituent building blocks (formed by molecules, molecular assemblies, or anisometric particles) (*49*) caused by chiral additives translates to the bulk, thereby facilitating the detection, measurement, and visualization of chirality on length scales easily accessible by optical microscopy between crossed polarizers.

**Fig. 1.**
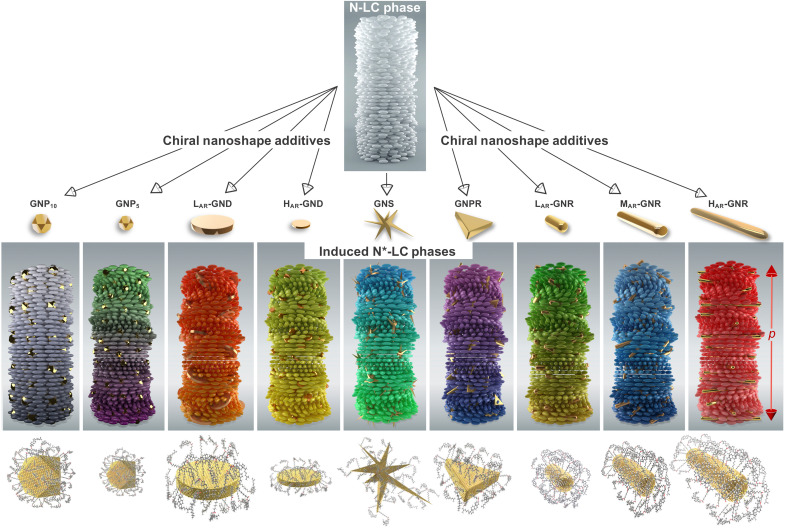
Nanoshapes and models of dispersions in N-LC medium. 3D models schematically showing the shape, surface modification, and the N*-LC phase induced by the dispersion of the cholesterol-thiol–capped nanoshapes into an achiral N-LC host (left-to-right): gold nanoparticles (GNP_10_ and GNP_5_ with core diameters of *d* ~ 10 nm and *d* ~ 5 nm, respectively), low– and high–aspect ratio gold nanodisks (L_AR_-GND and H_AR_-GND), gold nanostars (GNS), GNPR, and gold nanorods with low, medium, and high aspect ratio (L_AR_-, M_AR_-, and H_AR_-GNR); colors in the 3D models indicate anticipated (potential) changes and ranges in the measured helical pitch, *p*.

**Fig. 2.**
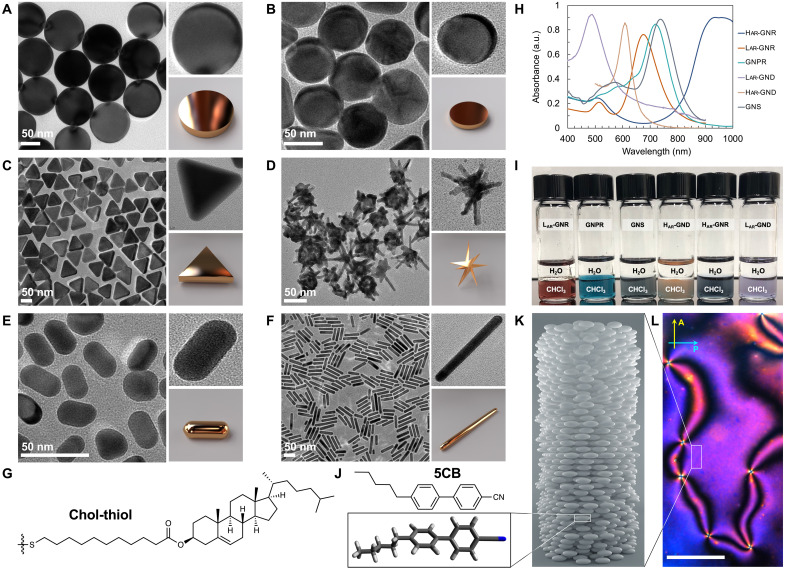
Selection of cholesterol-capped gold nanoshapes and achiral N-LC host. Transmission electron microscopy (TEM) images of (**A**) CTAC-coated L_AR_-GND, (**B**) CTAC-coated L_AR_-GND, (**C**) CTAC/CTAB-coated GNPR, (**D**) TX-100–coated GNS, (**E**) CTAB-coated L_AR_-GNR, and (**F**) CTAB-coated H_AR_-GNRs. (**G**) Chemical structure of the cholesterol-thiol ligand with C11-tether. (**H**) Vis-NIR spectra of the cholesterol-thiol–capped nanoshapes recorded in *n*-hexane. (**I**) Photograph showing vials of the nanoshapes dispersed in the organic layer of the binary solvent system CHCl_3_/H_2_O after ligand exchange. (**J**) Chemical structure and energy-minimized conformation of the achiral N-LC host 5CB. (**K**) Model of the N-LC phase and (**L**) polarized optical photomicrograph (crossed polarizer P and analyzer A) of the Schlieren texture of 5CB near room temperature (25°C) taken upon cooling from the isotropic liquid phase (showing alternating defects of strength +1 and −1); scale bar, 50 μm. See section S3 for additional TEM images of the precursor, surfactant-capped gold nanoshapes (fig. S2), vis-NIR spectra (fig. S3), and solution CD spectra of the cholesterol-thiol–capped gold nanoshapes (fig. S4).

To understand how the geometry of a chiral object affects chirality transfer, our approach focuses on the transmission and amplification of chirality from colloidal gold nanomaterials differing in shape, aspect ratio, and overall dimensions to an achiral N-LC medium. A combination of noncorrelated experimental and geometrically derived data will then reveal how chirality transfer efficacy depends on the geometry of well-defined chiral gold nanoshapes. The set of nanoshapes selected for this study includes gold nanoprisms (GNPR), nanodisks, polyhedral (quasi-spherical) nanoparticles, nanostars, and nanorods differing in aspect ratio (Fig. 1). These nanoshapes are monolayer capped with identical chiral ligands, i.e., a cholesterol-thiol derivative (Fig. 2), with the chiral cholesterol moieties amply distanced from the nanoshape surfaces by an aliphatic tether to avert chirality transfer to the plasmonic nanostructure itself.

The values of the experimentally determined helical pitch, *p*, of the induced N*-LC phase will then be used to calculate the helical twisting power [β*_w_*, defined as β_w_ = 1/(*p* ∙ *w*_Ligand_ ∙ *r*), in which *p* is the helical pitch, *w*_Ligand_ the weight fraction of the chiral ligand, and *r* the enantiomeric purity] of the well-dispersed nanoshapes (fig. S1).

The trends of the molar helical twisting power, β_mol_ = 1/(*p* ∙ *x*_Ligand_ ∙ *r*), in which *x*_Ligand_ is the mole fraction of the chiral ligand (*50*), present an experimental measure for chirality transfer efficacy to compare the independently calculated *G*_max_ and *G*_max_*S*^*XN*^ values. β_mol_ data are based on the actual number of chiral cholesterol molecules in each system and provide a more accurate platform to quantify individual helical twisting power values. Initial data for a small subset of these nanoshapes, GNP_10_, GNP_5_, and medium aspect ratio gold nanorods (M_AR_-GNR) revealed preliminary yet unusual trends. Between the two GNPs, GNP_10_ with the larger core diameter is noneffective in transmitting chirality to the N-LC with no indication of an induced N*-LC phase (*p* → ∞, the phase appeared achiral at all concentrations of GNP_10_) resulting in β_mol_ = 0 μm^−1^ (*40*). In contrast, GNP_5_ with the smaller core diameter was notably more effective, even more effective than the free organic chiral cholesterol derivative (β_mol_ = −22 μm^−1^; the negative sign indicating an induced left-handed N*-LC phase), inducing a tighter *p* at one order of magnitude lower number of chiral molecules as indicated by β_mol_ = −178 μm^−1^ (*40*).

Desymmetrization of the quasi-spherical GNP to elongated GNR such as M_AR_-GNR with a core aspect ratio of AR = 4.3 (with a transversal cross section of *d* = 10 nm matching the diameter of GNP_10_) ultimately led to another order of magnitude higher chirality transfer efficacy with β_mol_ = −1064 μm^−1^ as one of the highest values ever recorded (*40*). Moreover, this considerable amplification of the chirality transfer efficacy by M_AR_-GNR allowed us to compare the efficacy of a series of axially chiral binaphthyl-thiol ligands differing only in the length of a nontethered aliphatic chain in the 2′-position (with ∣β_mol_∣ values for each set of enantiomers ranging from 190 μm^−1^ to a record ~1300 μm^−1^) (*50*). Setting the stage for the current study, trends of ∣β_mol_∣ for these two nanoshapes, the GNP and M_AR_-GNR, set to be proportional to the chirality, closely followed the trends of the independently calculated, purely geometrical values for the average chirality index, $G_{\text{oa}}^{a}$, while adopting coarse-grained (CG) representations of the quantum-mechanically geometry-optimized cholesterol ligand molecules (see the Supplementary Materials and Methods section).

### Dependency of chirality transfer efficacy on the nanoscale shape

A naïve picture to draw from these preliminary data is to assume that one merely needs to increase the aspect ratio of a given shape, such as extending a quasi-spherical nanoscale polyhedron to a nanorod; furthermore, the shape of achiral host and chiral guest should be similar for efficacious chirality transfer and amplification. Simply put, an achiral host phase composed of ellipsoidal-like N-LC molecules doped with a chiral ligand–capped nanorod should give rise to cooperativity between host and guest, during which the chiral nanorod facilitates its own helical distortion in the induced elastic N*-LC medium. This notion found support from freeze-fracture transmission electron microscopy (TEM) experiments showing a clear helical arrangement of the M_AR_-GNR in the induced N*-LC phase with the distance between them matching the calculated particle-particle distance (*D*_P−P_) assuming well-dispersed GNRs. Cooperativity was further supported by anomalous trends in the plots of the inverse helical pitch (*p*^−1^) versus the concentration of the M_AR_-GNR in the N-LC host. Rather than increasing and reaching a plateau, a highly unusual, sudden, and sharp increase in the slope was observed in these plots when a certain threshold concentration [about 0.5 weight % (wt %)] was reached (*40*).

### Synthesis and characterization of gold nanoshapes

To more clearly delineate how the shape and AR of a chirality nanocarrier affect the efficacy of chirality transfer, we significantly extended the range of shapes and sizes to a total of nine different plasmonic nanoshapes with identical chiral surface chemistry. The synthesis and characterization of the additional nanoshapes—GNPR, gold nanostars (GNS), GNR with low and high aspect ratio (L_AR_-GNR and H_AR_-GNR), and small and large gold nanodisks (H_AR_-GND and L_AR_-GND)—is provided in sections S1 and S2. Representative TEM images of the precursor and visible and near infrared (vis-NIR) spectrophotometry data of the cholesterol-thiol–capped nanoshapes are shown in Fig. 2.

Analysis of the TEM images revealed that the purified and isolated nanoshapes feature narrow size and AR distributions (Table 1, fig. S2, and tables S1 to S6). The recorded vis-NIR spectra showed the characteristic hypsochromic or bathochromic shifts of surface plasmon resonance (SPR) bands expected for the various nanoshapes after the displacement of the surfactant layers by the cholesterol-thiol ligands (*50*). The H_AR_-GNR, L_AR_-GNR, and GNS displayed bathochromic shifts of the transversal SPR bands after displacement of cetyltrimethylammonium bromide (CTAB) and Triton X-100 (TX-100) with the cholesterol-thiols; the L_AR_-GND (*51*), H_AR_-GND, and GNPR showed a hypsochromic shifts after cetyltrimethylammonium chloride/bromide (CTAC/CTAB) mixed bilayers were displaced by the cholesterol-thiol ligand shell (fig. S3). Solution CD spectra of the gold nanoshapes recorded in *n*-hexane, as reported previously (*40*), showed CD signals exclusively in the ultraviolet (UV) spectral region between 200 and 300 nm corresponding to the cholesterol molecules tethered to the ligand periphery (fig. S4), supporting the argument that chirality transfer to the gold core is mute.

**Table 1.** Nanoshape dimensions. Dimensions and aspect ratios, AR, of the gold nanoshapes. For comparison, the aspect ratio of 5CB is AR_5CB_ = 4.

**Nanoshape**	**Diameter (*d*),** **height (*h*), length** (*l*)/(nm)*	**AR (no ligand** **shell)**	***AR* (+ ligand** **shell)^†^**
GNP_5_	5.5 (*d*)	1.0	1.0
GNP_10_	10.0 (*d*)	1.0	1.0
L_AR_-GNR	25.0 × 15.2 (*l* × *d*)	1.7	1.4
M_AR_-GNR	43.0 × 10.0 (*l* × *d*)	4.3	3.2
H_AR_-GNR	87.0 × 12.5 (*l* × *d*)	8.5	6.1
L_AR_-GND	79.0 × 21.6 (*d* × *h*)	3.7	3.8
H_AR_-GND	45.0 × 7.4 (*d* × *h*)	6.1	4.8
GNS	core: 60.0 (*d*); spikes: 69.0 (*l*)	–^‡^	–^‡^
GNPR	50.0 × 12.5 (*l* × *h*)^§^	5.3	3.5

*Determined by TEM image analysis (see section S3.1).

†Molecular length of the cholesterol-thiol ligand from energy-minimization *l* = 2.54 nm.

‡No aspect ratio was calculated.

§Assuming an equilateral triangle (with all sides of equal length, *l*) as the base shape.

To calculate each chiral molecule’s contributions toward chirality transfer to the achiral N-LC host, the average number of cholesterol-thiol molecules on the nanoshape surfaces needed to be determined as precisely as possible. Thus, thermogravimetric analysis (TGA) was used to analyze the monolayer coverage on the gold nanoshapes with the cholesterol-thiol ligands. The data showed close agreement with calculated numbers acquired, as reported previously (*40*, *50*), using a geometric model for each nanoshape (section S3, fig. S5, and table S7). Minor deviations between experimental weight loss values determined by TGA and values obtained from the calculations are due to inhomogeneities in size, AR, and ligand shell density (i.e., inhomogeneities in the ligand shell coverage). These inhomogeneities are not accounted for in the idealized geometric calculations.

### Cross-polarized light optical microscopy—Pitch measurements

**Fig. 3.**
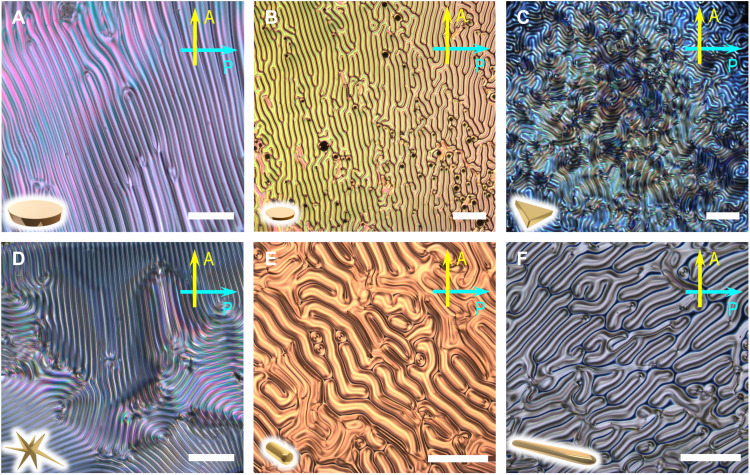
Fingerprint textures in N*-LC films—Homeotropic anchoring conditions. Polarized light photomicrographs (crossed polarizer P and analyzer A) of the N*-LC phase induced by the addition of 0.5 wt % of each nanoshape (schematically shown in the bottom left corner of each image) in 5CB at 25°C on cooling from the isotropic liquid phase in sandwiched glass cells treated to favor homeotropic anchoring conditions: (**A**) L_AR_-GND (cell gap = 50 μm), (**B**) H_AR_-GND (cell gap = 30 μm), (**C**) GNPR (cell gap = 20 μm), (**D**) GNS (cell gap = 20 μm), (**E**) L_AR_-GNRs (cell gap = 30 μm), and (**F**) H_AR_-GNRs (cell gap = 20 μm); scale bars, 50 μm. POM photomicrographs of 5CB doped with other concentrations of some of the nanoshapes are collected in figs. S9 and S10. This includes Grandjean-Cano wedge preparations using plano-convex lenses, thin film preparations between glass slides treated to favor homeotropic anchoring and free surface preparations (see Materials and Methods).

5CB (phase sequence on heating: *Cr* 22.5°C *N* 35.0°C *Iso*) was chosen as achiral N-LC host as it permitted all experiments to be performed at room temperature. Dispersions of these nanoshapes covering a range of concentrations from 0.1 to 0.7 wt % in the achiral N-LC host 5CB (4-cyano-4′-pentylbiphenyl) were then analyzed by a complementary set of polarized optical microscopy (POM) techniques to measure *p* and calculate β_w_ and β_mol_. These methods included the Grandjean-Cano wedge technique using plano-convex lenses as well as preparations of glass cells treated to favor homeotropic anchoring (Fig. 3) and preparations with one free surface (top interface is N*-LC/air; section S4 and figs. S6 to S10) that allow the polarized light microscopic measurement of *p* (see Materials and Methods).

Figure 3 (A to F) shows POM images of the characteristic fingerprint textures of the induced N*-LC phase in sandwich cell preparations treated with polyimide alignment layers favoring homeotropic anchoring conditions. Homeotropic anchoring allows for determining *p* microscopically by the formation of these fingerprint textures where periodic distances of disclination lines between the bright and dark areas correspond to the helical half pitch, *p*/2, of the induced N*-LC phase. For the GNPR and GNS, inducing tighter *p* values, precise values for *p* were also obtained by injecting the induced N*-LC phase between a plano-convex lens of known curvature and a flat glass substrate, both treated with a rubbed polyimide favoring planar boundary conditions. This geometry produces Grandjean-Cano disclination lines (fig. S8). The concentric circular Cano lines in the N*-LC phase develop because of the rotary power of the N*-LC and the curvature radius, *r*, of the plano-convex lens. Table 2 provides an overview of all measured values for *p* and additionally shows that the average *p* values obtained from the fingerprint textures and the Grandjean-Cano lens experiments (where attainable) closely match. For the GNS and GNPR in 5CB, aggregation occurred at concentrations exceeding 0.6 wt %.

**Table 2.** Summary of experimental and calculated data. Weight fraction (*w*_Ligand_), mole fraction (*x*_Ligand_), helical pitch, *p* values, helical twisting power (β_w_), molar helical twisting power (β_mol_), average particle-particle distance (⟨*D*_P−P_⟩), and number of nanoshapes per full helical pitch of the chiral cholesterol-thiol–capped nanoshape additives in 5CB based on a constant weight fraction of 0.5 wt %.

**Nanoshape**	***w*_Ligand_ × 10^−3^**	***x*_Ligand_ × 10^−4^**	*p* (μm)*		**β_w_^†^ (μm^−1^)**	**β_mol_^†‡^ (μm^−1^)**	**⟨*D*_P−P_⟩ (nm)**	**Nanoshapes** **per full pitch**
			**Free surface**	**Lens or wedge**				
GNP_5_ (*48*)	1.05	4.40	12.0	13.0	−76.0 ± 3.8	− 178 ± 9.0	24.0	52
GNP_10_ (*48*)	0.77	3.30	–^§^	–^§^	–^||^	–^||^	53.2	–^||^
L_AR_-GNR	1.10	4.68	50.0	–^¶^	−23.9 ± 1.2	−53.4 ± 2.7	42.8	1190
M_AR_-GNR (*50*)	2.00	8.50	1.3	1.1	−455 ± 23	−1064 ± 53	47.6	25
H_AR_-GNR	1.00	4.26	130.0	–^¶^	−7.70 ± 0.4	−18.1 ± 1.0	53.0	2452
L_AR_-GND	1.40	5.96	45.0	–^¶^	−15.6 ± 0.8	−37.3 ± 1.9	84.5	537
H_AR_-GND	1.35	5.75	40.0	–^¶^	−20.0 ± 1.0	−43.1 ± 2.2	59.4	666
GNS	1.50	6.38	15.0	14.8	−41.8 ± 2.1	−105.1 ± 5.3	106	141
GNPR	1.25	5.32	9.0	8.4	−94.6 ± 4.8	−216.0 ± 11	58.4	155
Ligand								
Chol-thiol	5.0	21.2	58.0	58.0	−3.5 ± 0.2	−8.1 ± 0.4	14.6^#^	–

*The values of *p* obtained by the different measurement setups (Grandjean-Cano plano-convex lens, free surface, or homeotropic anchoring) are, as reported previously, in close agreement. Accuracy is ±0.3 μm.

†The negative sign indicates that the induced N*-LC phase is left handed as established by contact preparations with a known left-handed N*-LC material, cholesteryl oleyl carbonate (COC). Cooperativity among the nanoshapes leading to enhanced values for β_w_ and β_mol_ was again observed, supported by the anomalous trends in the plots of the inverse helical pitch (*p*^−1^) versus the nanoshape concentration (fig. S12); the standard deviations partly accounted for this.

‡β_mol_ is the helical twisting power value when the dimensionless mole fraction (*x*_Additive_) of the chiral additive is used. For the current dataset, *x*_Additive_ = *x*_Ligand_ because the nanoshapes are intrinsically achiral. To determine *x*_Ligand_, the experimental TGA data (see table S7) were used.

§The helical pitch was too large to be measured and the sample appeared as an achiral N-LC phase in all experiments.

||Since *p* was too large to be measured, these values could not be calculated and would signify β_w_ and β_mol_ = 0 μm^−1^.

¶The curvature of available plano-convex lenses did not allow for measurement of these larger *p* values; only values from free surface and preparation with homeotropic anchoring conditions were used.

#*D*_P–P_ here refers to the average distance between well-dispersed cholesterol disulfides used as chiral additive in 5CB.

Considering the measured helical pitch values at a given weight faction of 0.5 wt % in 5CB, nanoshapes can be sorted into five categories. The M_AR_-GNR induce, by far, the tightest *p* of ~1.2 μm in 5CB and H_AR_-GNR, a pitch that is about two orders of magnitude larger with *p* = 130 μm. The GNPR, GNS, and GNP_5_ belong the category with values of *p* between 9 and 15 μm, and the two GNDs and L_AR_-GNR represent the fourth category with *p* ranging from 40 to 50 μm; GNP_10_ did not show any chirality transfer (or chiral textural features in any of the used methods), as reported earlier, and therefore, *p* ⟶ ∞. Without precise knowledge of the exact number of chiral cholesterol molecules involved, these data may indicate little accordance with the aforementioned naïve picture.

### Thin-film induced CD spectropolarimetry

Thin-film induced CD (ICD) spectropolarimetry is another technique to examine the chiroptical activities induced by the chiral nanoshapes into an achiral self-assembled medium. Thin-film preparations between two quartz plates with a nominal cell gap of ∼20 μm were rotated in 45° intervals from 0° to 315° (in the plane normal to the light beam) to differentiate circular CD absorption from linear dichroism and birefringence (*52*). The averaged ICD spectra (average of the spectra collected at eight sample rotation angles) displayed prominent CD reflection or absorption signals for 5CB doped with the various nanoshapes. Reflection CD signals for 5CB doped with the GNPR, GNS, L_AR_-GNR, GNP_5_, and M_AR_-GNR start at a wavelength of ∼320 nm (absorption edge of 5CB) (*52*) and cover virtually the entire visible part of the spectral range, thereby signifying the tighter *p* values seen in the POM studies. Absorption CD spectra for the L_AR_-GND, H_AR_-GND, and H_AR_-GNR with maxima centered around 320 nm are due to selective reflection from the pitch of these N*-LC films (section S5 and fig. S11). These sharp but less intense CD absorption bands indicate larger *p* values also seen by POM. The negative sign of all CD reflection and absorption signals is due to a consistently left-handedness of the induced N*-LC phase further supported by contact preparations with a known left-handed N*-LC material. With POM and CD providing relative values and data, we next calculate β_w_ and β_mol_ as measures for the chirality transfer efficacy as well as the average distance between nanoshapes *D*_P–P_ (Table 2 and section S7.1) in the induced N*-LC bulk as a measure for the correlation lengths between chiral inducers to induce a constant *p* throughout the N*-LC bulk.

### Calculation and comparison of β_w_, β_mol_, and *D*_P−P_

To correctly assess the effects of size, shape, and AR (i.e., the geometric anisotropy) on the magnitude of chirality transfer efficacy, we compare the calculated β_w_, β_mol_, and *D*_P–P_ summarized in Table 2. As expected, even with the exact number of chiral molecules included in the calculation of β_mol_, the earlier established categories hold. M_AR_-GNR and H_AR_-GNR give the highest and lowest absolute values of ∣β_mol_∣, respectively. The L_AR_-GND, H_AR_-GND, and L_AR_-GNR form the category with the lower midrange β_mol_ values; the GNPR, GNS, and GNP_5_ the category with the higher midrange values; and GNP_10_ is set to β_mol_ = 0 μm^−1^.

Apart from GNP_10_, all the cholesterol-thiol–capped gold nanoshapes continue to reinforce the argument that affixing a chiral molecule to a gold nanocarrier results in chirality amplification, vis-à-vis induction of a N*-LC phase, when comparing the values of the quantitative measures β_w_, β_mol_, and *D*_P–P_ to those calculated for the free cholesterol thiol (i.e., the chemically stable disulfide) molecule in 5CB (Table 2).

At a constant weight fraction, the larger and therefore heavier nanoshapes result in larger values for *D*_P−P_. Thus, with respect to *D*_P−P_ among all nanoshapes, the GNS give the largest distance among all tested nanoshapes (*D*_P−P_ ~ 106 nm), exemplifying their uniquely enhanced ability to transfer chirality over large distances, despite a comparatively lower value of ∣β_mol_∣ in comparison to the M_AR_-GNR, which induce by far the tightest helical pitch of *p* ~ 1.2 μm. GNP_5_, as the smallest nanoshape, shows as anticipated the lowest *D*_P−P_. Knowing *p* and *D*_P−P_ (assuming well-dispersed nanoshapes), we also calculated the number of nanoshapes in one full pitch of the induced N*-LC helix (Table 2). These values in conjunction with β_mol_ give a first indication about how the geometry of the dispersed nanoshapes affects chirality transfer and amplification. However, to better understand how geometry affects the chirality of these nanoshapes, we next consider calculations of the maximum chirality indicator $|G_{\text{oa},\text{max}}^{a}|$ and the shape compatibility–corrected $|G_{\text{oa},\text{max}}^{a}S^{\mathrm{XN}}|$.

### Chirality indices and isoperimetric ratios

We quantify the chirality of our nanoparticle-ligands with the help of the average or maximum chirality indicators $\langle G_{\text{oa}}^{a}\rangle$ or $|G_{\text{oa},\text{max}}^{a}|$, which were previously validated and used for small molecules, proteins (*41*), and a limited number of gold nanoshapes (GNP_5_, GNP_10_, and M_AR_-GNR) (*40*, *50*), but recently also for systems as diverse as the change in winding direction of erodium awns (*53*). These indices depend only on geometric information, i.e., in this case, the position and orientation of the cholesterol-thiol ligands with respect to the nanoshape frame, and thus indirectly on the shape and size of the nanoshape

$$G_{\text{oa}}^{a}=\sum_{P}\{\begin{array}{ll} \frac{\left[(r_{\text{ij}}\times r_{\text{kl}})\cdot r_{\text{il}}](r_{\text{ij}}\cdot r_{\text{jk}})(r_{\text{jk}}\cdot r_{\text{kl}}\right)}{n{\left(r_{\text{ij}}r_{\text{jk}}r_{\text{kl}}\right)}^{2}r_{\text{il}}} & \text{if}\;i<j<k<l\;\epsilon \;[1,n]\\ & 0\;\text{otherwise} \end{array}$$ (2)

in which *n* is the total number of atoms (or beads) and ***r***_ij_ is the vector distance between any two atoms i and j (subscript oa stands for overall, the index of summation, *P*, for the permutations of the beads i, j, k, and l). Given a chiral solute *X* (in this case, the nanoshapes) in an achiral nematic phase *N*, the pitch *p* of the induced N*-LC phase is inversely proportional to the chiral dopant concentration, $c_{X}^{*}$, and to the helical twisting power (β*^XN^*)

$$p\propto 1/(\beta^{\mathrm{XN}}c_{X}^{*})$$ (3)

We do not consider changes in $c_{X}^{*}$ or changes in temperature since these were constant from one nanoshape to another in the experimental systems. We further assume the isotropic to nematic phase transition temperature, *T*_NI_, to be the same in each case, and that *p* was measured at the same temperature below *T*_NI_, which is congruent to experiment conditions, in which minute quantities of the dispersed nanoshapes did not alter *T*_NI_ with respect to the neat N-LC. Thus, we assume the order parameter ⟨*P*_2_⟩ of the induced N*-LC phase to be identical in each system. The orientational order of the nematic system formed by rod-like molecules with molecular axis ***u***_i_ and local preferred direction (director) ***d***, i.e., 〈*P*_2_〉 = 〈3 (***u***_i_ · ***d***)^2^ − 1〉/2 is to a first approximation a nearly universal function of the reduced temperature *T*/*T*_NI_.

We use $|G_{\text{oa},\text{max}}^{a}|$ to examine whether changing the number, position, and orientation of the ligands on the surface of the nanoshapes differing in size, shape, and AR leads to variations in chirality similar to the performed experiments, with the usual assumption that β_w_ and β_mol_ are proportional to the chirality ($\beta_{x}\propto |G_{\text{oa},\text{max}}^{a}|$). To do this, we adopted a CG representation of the cholesterol-thiol ligands (fig. S13) and distributed a certain number of these ligands (20 to be exact) on the surface of each nanoshape with relative dimensions corresponding to the experimental data (fig. S14).

In more detail, the nanoshapes were divided in three parts: top, middle, and bottom. CG ligands were placed, not randomly, but in a uniform fashion such that similar ligand placements were reproduced for all nanoshapes (across these three parts). We assumed that nanoshape interactions and aggregation are negligible. For each nanoshape, *m* + 2 positions have been selected as possible “bonding sites.” Out of these, *m* positions are chosen to generate a single configuration. The CG ligands, represented by a set of beads (see fig. S13), point straight outward from the nanoshape at specific values of an angle θ (θ = 0° or θ = 60°; see fig. S14) between the ligand “main axis” shown in fig. S13 and the normal to the plane tangent to the nanoshape surface at that specific point (fig. S14). Choosing *m* unordered outcomes out of *m* + 2 possibilities leads to $(\begin{array}{c} m+2\\ m \end{array})=\frac{(m+2)!}{m!2!}=\frac{\left(m+2)(m+1\right)}{2}$ configurations. Considering the two possible values of θ for the top, middle, and bottom part, this gives a total of (*m* + 2)(*m* + 1) × 2^2^ possible combinations for each nanoshape studied. In practice, each nanoshape was decorated with *m* = 20 CG ligands, which leads to 231 configurations and a total of 231 × 23 = 1848 combinations, which, irrespective of size, maps out the nanoshape. For each of them, using Eq. 2, we have calculated the range of chirality values and obtained their maximum average values (see Materials and Methods).

The positions of the CG ligands capping the nanoshape have been chosen following, as far as possible, the same procedure for each nanoshape (fig. S14). All GNR shapes have one possible ligand at the end points (top and bottom) and the remaining 20 positions were situated along the GNRs’ long axes, at five different heights and at four different positional angles (0°, 90°, 180°, and 270° observing the center cylinder from top). The GNPR shape has possible attachment points at the top (2) and at the bottom (2) of the central part, plus three ligands (placed at three different heights) at the corners and at the center of each side. The GNS shape has four possible ligand bonding points in the central spheres plus three at the end of each spike. Disks can have ligands placed in columns (three at different heights) placed at four different angles; both on the top and at the bottom, there are five possibilities for ligands to bind, one at the center and four around it. Last, for the GNP shape, we have chosen one point, labeled top pole, and one CG ligand is placed there, and another one at the opposite pole, labeled bottom. The remaining 20 positions have been placed around the quasi-spheres in four columns (at the previously listed angles).

In contrast to this approach, we also attempted to choose a varying number of CG ligands so as to keep the density of ligands constant. However, this approach leads to chirality data that are strongly related to the number of CG ligands placed on each nanoshape. With the chirality index increasing linearly with the number of CG ligands (trend preserving), this creates additional challenges to compare nanocarriers differing in size and shape. Thus, maintaining a constant number of CG ligands forces the overall chirality index to solely depend only on the shape of the nanocarrier, thereby closely capturing the essence of the experimental system.

Hence, each nanoshape was decorated with the same number of CG ligands to avoid effects on the overall chirality because of the different number of chiral ligand molecules. This procedure is inevitably arbitrary, at least to some extent, lacking experimental information of the exact distribution of ligands. However, the number of possible combinations leads to an overall homogeneous coverage of each nanoshape, and in our experience provides an adequate number of ligand arrangements, while still being tractable in terms of computer times.

Our approach to use $|G_{\text{oa},\text{max}}^{a}|$ is further supported by the fact that the helical twisting power data are calculated from experimental pitch measurements, which are particularly sensitive to the highest rather than average chirality values (particularly for a system containing various contributions of different chirality). To further confirm the validity of this assumption, we recalculated previously published chirality index data (*50*) with this maximum parameter (instead of the average), and the trends are in very good agreement with the experimentally derived β_w_ and β_mol_ data (fig. S15).

**Fig. 4.**
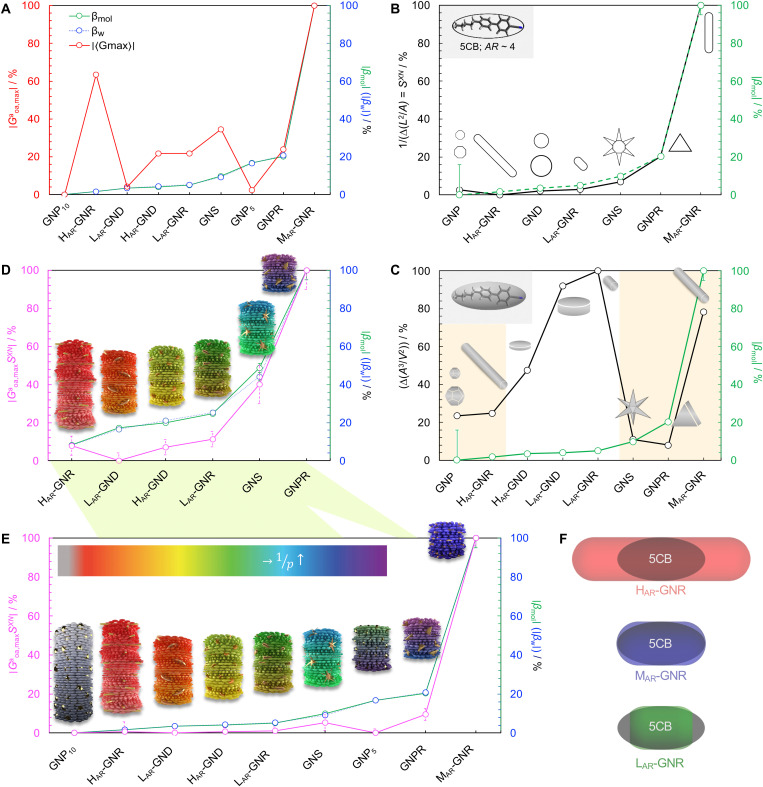
Comparison of $|G_{\text{oa},\text{max}}^{a}|$, isoperimetric ratios (*S^XN^*, IPR_3D_), and $|G_{\mathrm{oa},\text{max}}^{a}S^{\mathrm{XN}}|$ with experimentally determined or derived values for β_w_ and β_mol_. Trends of (**A**) maximum chirality indicator $|G_{\text{oa},\text{max}}^{a}|$ in comparison to ∣β_w_∣ and ∣β_mol_∣; for the calculated values of β_w_ and β_mol_, see Table 2. (**B**) $|\frac{1}{\text{∆}({\text{IPR}}_{2D})}|$and (**C**) $|\frac{1}{\text{∆}({\text{IPR}}_{3D})}|$ each in comparison to ∣β_mol_∣ [yellow shaded areas in (C) signify some correlation in trends]—for values of IPR_2D_ and IPR_3D_, see section S7.4; values for IPR_2D_ and IPR_3D_ use the adjusted AR values considering the thickness of the ligand shell as collected in Table 1 and in the section S7.3.1 (fig. S16). (**D** and **E**) Shape compatibility–corrected maximum chirality indicator $|G_{\text{oa},\text{max}}^{a}S^{\mathrm{XN}}|$ in comparison to ∣β_w_∣ and ∣β_mol_∣; (D) shows the current subset of previously unpublished nanoshapes. Models of the induced N*-LC phase show a complete 360° rotation of the local director leading to a tighter pitch from left to right (according to the used color scheme, if the reflection notch range—depending on the nanoshape concentration in 5CB—of the induced N*-LC phase was in the visible) depending on the size, shape, and AR of the suspended nanoshape (for GNP_10_: *p* ⟶∞). (**F**) Visualization demonstrating the varying degree of shape compatibility among the series of GNRs with the 2D projection of 5CB scaled in size to match each GNR’s width.

To compare these results with the experimental data collected in Table 2, we have converted the β_w_ and β_mol_ values into percentages. The trends of ∣β_w_∣ and ∣β_mol_∣ are quite similar, i.e., these two quantities largely overlap, and we have plotted these experimental data against the $|G_{\text{oa},\text{max}}^{a}|$ calculation results, which were also converted into percentages (Fig. 4A). One notices that the trend of the computed chirality index $|G_{\text{oa},\text{max}}^{a}|$ is in reasonable agreement with the experimental trends of ∣β_w_∣ and ∣β_mol_∣. In particular, the variation of chirality with size, shape, and AR, with the only exception of H_AR_-GNR, is well reproduced, notably without any fitting parameter. Significantly, no specific material parameters were introduced for the nature of the gold nanomaterials. Thus, these results suggest that the origin of the chiral amplification effects, governed by the geometry of the nanoshape, is in some form connected to the chiral ligands forming a network with a spatial distribution mimicking the nanoshape. Hence, all ligands act together in a correlated way, augmenting, as seen experimentally, the overall chirality.

Next, we consider a reinforcing helical arrangement of the nanoshapes, self-induced by the helical distortion of the N*-LC phase as described for the M_AR_-GNR (*40*). This reinforcement would be particularly critical for nanoshapes that induce and maintain such a helical distortion over larger distances, i.e., larger *D*_P–P_.

The second term in Eq. 1 is the shape complementarity, and we base a measure for this initially abstract concept using the IPR_nD_. Each of the nanoshapes has its own unique geometries while sharing the same chemical composition. These ratios are dimensionless constants that are invariant under spatial transformations (i.e., translation, rotation, and scaling), such that proportionally increasing or decreasing the dimensions of the nanoshapes does not influence their values; this effect can be observed from the GNP data for IPR_nD_ and the GND data for IPR_2D_. However, manipulating the AR of geometrically similar shapes will alter their respective IPR values. For example, each of the GNRs has a unique AR (Table 1) that characterizes its perimeter-to-area (2D), or surface area-to-volume (3D), ratio (see section S7.3 and tables S8 to S10).

Determining the dimensions of the most practical model is not immediately apparent, and both IPR_2D_ and IPR_3D_ are considered. Considering an idealized N*-LC helical structure as a stack of virtual planes twisted with respect to each other, it seems reasonable to assume that the compliance between solute (nanoshape) and nematogen shape concerns the projection on the (*xy*) plane of the two, i.e., the chiral ligand–capped nanoshapes and the mesogen molecules (along and across the longer axes in the 2D projections). Projecting the profile of a 3D structure onto a 2D surface and comparing the resulting 2D shape to that of the N-LC host 5CB may clarify whether a nanoshape’s β_mol_ value is enhanced itself by the similarity of the geometry of the nanoshape with the geometry of the host molecules. These projections, or plane curves, of the 2D shapes are local parameterization of the nanoshape on the host nematic; because of the parallax error, the 3D nanoshapes look different at each point in the LC reference frame. Essentially, a local arrangement of N-LC molecules (or a plane of them) can “see” a 2D parameterization of the 3D object at every point on the shape; errors due to parallax occur around edges, points, and arcs). Thus, we define the 2D shape compatibility factor, *S^XN^*, between *X* and *N* (here 5CB), as the absolute value of the inverse difference between the IPR_2D_ of each nanoshape and the IPR_2D_ of 5CB (Eq. 4)

$$S^{\mathrm{XN}}\sim |\frac{1}{\Delta (\text{IP}R_{2D})}|$$ (4)

i.e., the closest match between their respective 2D shapes set to 100% (table S8). The validity of pursuing this 2D approach is then underpinned by a comparison of the data, which reveal a closer match between $|\frac{1}{\Delta (\text{IP}R_{2D})}|$ and the experimentally derived ∣β_mol_∣ values (Fig. 4B).

In contrast, $|\frac{1}{\Delta (\text{IP}R_{3D})}|$ only shows limited correlations to the experimentally acquired ∣β_mol_∣ values (Fig. 4C). Thus, the 5CB host molecules experience chirality transfer from the 2D projection of the suspended nanoshapes, i.e., from the distribution of chiral molecules on the nanoshape surface they are in direct contact with. Furthermore, this shape coupling or compatibility factor *S^XN^* becomes most relevant when the shape of *X* is changing within a series of solutes in the same *N*. In Fig. 4F, we sketch the shape compatibility for the subset of the nanorods, H_AR_-, M_AR_-, and L_AR_-GNR that best demonstrates the concept and utility of introducing *S^XN^*.

Plotting the shape compatibility–corrected $|G_{\text{oa},\text{max}}^{a}S^{\mathrm{XN}}|$against the experimentally obtained values shows very close correlation across almost all nanoshapes with respect to ∣β_w_∣ and ∣β_mol_∣ excluding only GNP_5_ (Fig. 4E and a subset showing the previously unpublished cholesterol-thiol capped nanoshapes in Fig. 4D), again all in the absence of any fitting parameter. For GNP_5_, the underestimated $|G_{\text{oa},\text{max}}^{a}|$ value (Fig. 4A) is not sufficiently compensated by *S^XN^*, thus resulting in a low $|G_{\text{oa},\text{max}}^{a}S^{XN}|$ value not reflecting the experimentally obtained values for ∣β_w_∣ and ∣β_mol_∣. Another explanation for the experimentally acquired higher ∣β∣ values for GNP_5_ could also be a chirality transfer from the chiral ligand shell to the GNP_5_ core that is not accounted for in the $|G_{\text{oa},\text{max}}^{a}S^{XN}|$ calculation. We have further checked the $|G_{\text{oa},\text{max}}^{a}S^{\mathrm{XN}}|$ and the *S^XN^* parameters against the ∣β_w_∣ and ∣β_mol_∣ data previously published, verifying that both parameters still describe the experimental results appropriately with the closest correlation again between the experimental helical twisting power and $|G_{\text{oa},\text{max}}^{a}S^{\mathrm{XN}}|$ (fig. S17), thereby verifying the validity of our approach in light of currently available experimental data.

In conclusion, our experimental data verify that the size and aspect ratio of chiral ligand–decorated nanoshapes affect in now-predictable ways the chiral perturbation induced in an achiral host medium given by an N-LC phase. Each of the surveyed gold nanoshapes capped with a cholesterol-thiol ligand outperforms the neat organic chiral molecules with respect to its ability and effectiveness to transfer chirality to the host medium. We firmly established a systematic correlation between purely geometric concepts of an independently calculated chirality indicator and experimental chirality transfer data. This methodology provides a broadly expandable tool to a priori predict and then experimentally confirm chirality transfer efficacy from an innate chiral object to an achiral medium. In the current experimental system, this opportunity is offered by measuring the induced helical pitch and calculating the helical twisting power in an induced N*-LC medium for essentially any possible nanoshape varying in size, shape, or AR, all without any need to alter or adjust the chemical nature of the chiral molecule decorating the nanoshape surface. The closest agreement between theory and experiment is achieved by introducing a shape-compatibility factor in the form of an inverse-difference between the isoperimetric ratios of nanoshape solute and host molecules as a coupling parameter for the pseudoscalar chirality index that is based upon the molecular conformation of the chiral ligand molecules forming the shell around the various nanoshapes. Whatever value of *p* that may be required can be zeroed in on by preselecting a suitable nanoshape, after calculating $|G_{\text{oa},\text{max}}^{a}S^{\mathrm{XN}}|$, with the appropriate ∣β_w_∣ or ∣β_mol_∣.

Our approach relies on some ad hoc assumptions, and, in principle, some other strategies could be developed to connect shape with twisting power. For instance, the importance of geometrical factors in determining chirality and phase behavior has been discussed by Dussi *et al.* (*54*) and Frezza *et al.* (*55*), where the authors detailed that a helical array of purely repulsive spherical beads can form chiral nematics and even more complex screw phases (*56*). However, we are not aware of any modeling of this type of chirality transfer, with hard chiral particles inducing a twist in nematics. A specific effective field approach to cholesteric induction from a chiral solute dissolved in a nematic is instead that originally proposed by Ferrarini *et al.* in (*57*). In their approach, the solute shape enters through the assumed tendency of a vector normal to the exposed surface elements of the solute molecules to align with the local director. The methodology has been applied with some success to a number of only low molar mass chiral molecules in nematics as reviewed in (*58*). However, applying these particular systems to rather different length scales as in the case of the nanoshapes described here seems thus far out of reach.

With the practical tools developed here at hand, the efficacy of chirality transfer as a geometric concept can now be theoretically (geometrically) predicted. A further extension of this concept could be used as an indirect measure (a quasi-indirect TEM experiment) to determine a nanomaterial shape and even shape distribution, if, as demonstrated here, a catalog of sizes, shapes, or ARs is available.

Once expanded to other systems and further reinforced by machine learning methods, such a straightforwardly applied geometric approach should provide a vital tool for experimentalists in many fields where chirality transfer is essential. These may include, among many others, the development of chiral nanomaterial catalysts for solid- or liquid-phase asymmetric synthesis and of chiral stationary phases indispensable for the chiral separation of pharmaceuticals. Furthermore, using such a predictive tool will enable a better understanding of chiral molecular recognition and amplification events (receptor-host or substrate-guest) occurring in all living systems and a source of transmission of biological homochirality as well as accelerate advances in chiral nanophotonics, metamaterials, and chiral actuators.

## MATERIALS AND METHODS

### Materials

Hydrogen tetrachloroaurate trihydrate (HAuCl_4_ · 3H_2_O, >99% purity; CAS. no. 16961) and *L*(+)–ascorbic acid (>99% purity; CAS. no. 36237) were purchased from Alfa Aesar. Silver nitrate (AgNO_3_, >99% purity; CAS. no. 209139), cetyltrimethyl ammonium chloride (20 wt % in H_2_O; CAS. no. 292737), sodium iodide (NaI, 99.5% pure; CAS. no. 383 112), sodium borohydride (NaBH_4_, >99% purity; CAS. no. 213432), hydroquinone (>99%; CAS. no. 123319), Triton X-100 (laboratory grade; CAS. no. 9002931), sodium hydroxide (NaOH, >99% purity; CAS. no. 1310732), and hydrochloric acid (HCl, 37%; CAS. no. 2 019 061 317) were obtained from Sigma-Aldrich. Hydrogen peroxide (H_2_O_2_, 30% in H_2_O; CAS. no. 211016) was obtained from BDH, and CTAB (>98% purity; CAS. no. 57090) and 5CB (>98% purity; CAS. no. C1550) from TCI America. Deionized water was Millipore pure water (resistivity: 18.1 megaohms or MΩ). PI-2555 was purchased from HD Microsystem and SE-1211 from Nissan Chemicals. Spherical spacers with different diameters were obtained from Dana Enterprise International Inc. Solvents used for the synthesis and purification were EMD Millipore grade, purified by PureSolv solvent purification system (Innovative Technology Inc.).

### Methods

For the characterization of the nanoshapes, TEM was performed using an FEI Tecnai TF20 TEM (200 kV), UV-visible and CD spectrophotometry used an OLIS 17 spectrophotometer (Olis), and a TGA Q500 (TA Instruments). Polarized light optical microscopy used an Olympus BX3 microscope equipped with an LTS 420E heating/cooling stage from Linkam Scientific Instruments.

### Synthesis

The synthesis of the cholesterol-disulfide used for all nanoshape ligand exchange reactions and the synthesis for the cholesterol-thiol surface–modified M_AR_-GNR (*40*), the polyhedral (quasi-spherical) gold nanoparticles (GNP_5_ and GNP_10_) (*48*), the precursor (surfactant-capped) low–aspect ratio GND (L_AR_-GND) (*51*), and the precursor (surfactant-capped) GNS were previously reported (*59*). The detailed synthesis procedures, including the ligand exchange, for the GNPR (*60*–*64*), high–aspect ratio gold nanodisks (H_AR_-GND) (*56*), L_AR_-GNR (*65*), and H_AR_-GNR (*62*) are provided in the Supplementary Materials. The average size distributions from the TEM images were obtained using ImageJ (*66*).

### Preparation of mixtures and helical pitch measurements

Admixing of the gold nanoshapes was achieved following a standard protocol as follows: Precise quantities were weighted into rigorously cleaned glass vials using an ultramicrobalance. Standardized solutions of each chiral nanoshape additive and N-LC host 5CB in a common organic solvent (purified CHCl_3_) were prepared, and the desired volumes of each solution were combined using calibrated Eppendorf pipettes and thoroughly mixed. Thereafter, the solvent was evaporated under a steady stream of nitrogen followed by mild vacuum. Homogeneous dispersions were ensured by mild, pulsed sonication at 25°C for 5CB, that is, with the mixtures in the induced N*-LC phase.

To measure *p* of the induced N*-LC phase, Grandjean-Cano wedge cell–based (*67*) convex lens measurements and preparations with one free surface and homeotropic anchoring conditions were used. Free surface measurements (*68*) used precleaned but untreated glass substrates favoring degenerate planar anchoring, onto which one drop of the N*-LC/nanoshape mixtures was placed. The top boundary is air, which favors homeotropic anchoring of N-LCs such as 5CB. Fingerprint textures were obtained in these preparations such that the distances between dark striations under crossed polarizers were measured using the imaging software calibrated via a microruler. For the lens measurement, substrates were fabricated by using precleaned glass substrates onto which polyimide PI2555 was spin coated, baked, and rubbed (40 times) to give strong planar anchoring. The same was performed on various plano-convex lenses. Substrate and lens were then assembled unidirectionally by placing a drop of the N*-LC mixture on the planar alignment favoring flat substrate and placing the lens on top. All measurements were done at 25°C. Additional methods used to determine *p* measured the distances between either birefringent or dark striations in cells prepared to favor homeotropic anchoring conditions. The fingerprint textures were obtained in homeotropic cells constructed using two cleaned glass substrates spin coated with polyimide SE1211 and baked to provide strong homeotropic anchoring. Cells were assembled with different cell gaps depending on the magnitude of *p*. After filling the cells with the N*-LC/nanoshape mixtures by capillary force, cells were sealed with epoxy adhesive.

### Calculations

More details about the calculation of the chirality indices are provided in the Supplementary Materials (section S7). This includes a comparison of $G_{\text{oa}}^{a}$ and $G_{\text{oa},\text{max}}^{a}$ using GNR capped with axially chiral binaphthyl-thiol ligands previously reported (*69*). For the calculation of the isoperimetric ratios, we assumed the following: For 5CB, an ellipsoidal shape with an aspect ratio of AR ~ 4 [*l* = 1.8 nm, *w*_eff_ = 0.45 nm (*70*)] was calculated (energy-minimized) assuming a conformation in the nematic phase with free rotation about the long molecular axis. For the GNP, depending on the polyhedral shape that can include a range of Platonic and Archimedean solids (*71*), the most energetically favored truncated octahedron was used (*72*). For more details, see section S7.

## Supplementary Materials

### This PDF file includes:

Detailed Methods Figs. S1 to S17 Tables S1 to S10
