# Supplementary material for: Effects of shape and solute-solvent compatibility on the efficacy of chirality transfer: Nanoshapes in nematics
Source: Sci Adv. 2022 Jan 26;8(4):eabl4385. doi: 10.1126/sciadv.abl4385 (PMC8791610; doi:10.1126/sciadv.abl4385)
Supplement: Supplementary file 1 — Detailed Methods Figs. S1 to S17 Tables S1 to S10 [file sciadv.abl4385_sm.pdf]

Science Advances
AAAS

Supplementary Materials for

Effects of shape and solute-solvent compatibility on the efficacy of chirality transfer: Nanoshapes in nematics

Ahlam Nemati, Lara Querciagrossa, Corinne Callison, Sasan Shadpour, Diana P. Nunes Gonçalves, Taizo Mori, Ximin Cui, Ruoqi Ai, Jianfang Wang, Claudio Zannoni*, Torsten Hegmann*

*Corresponding author. Email: claudio.zannoni@unibo.it (C.Z.); thegmann@kent.edu (T.H.)

Published 26 January 2022, *Sci. Adv.* **8**, eabl4385 (2022)
DOI: 10.1126/sciadv.abl4385

This PDF file includes:

Detailed Methods
Figs. S1 to S17
Tables S1 to S10

S1. DETAILED METHODS

S1.1. BASIC SYNTHESIS EQUIPMENT

- Round hotplate stirrer with corresponding temperature controller 6.81 in × 10.9 in, 100 – 1,400 rpm (Heidolph 036110519)
- Disposable borosilicate vials with plastic caps (VWR, Cat.no.66011-143)
- Micropipettes with disposable tips (ranges: 2 μL – 1,000 μL; Eppendorf)
- PP Disposable microcentrifuge tubes, capacity 2 mL (VWR, cat.no.20170-170)
- Glassware (Chem Glass)
- PVC disposable plastic syringes (VWR, volume range: 1 – 10 mL)
- Precleaned substrates, glass (Loveland, CO)
- Ultrasonic bath (Branson 1510)
- Carbon-coated TEM grids (400 mesh, TED PELLA, Inc.) for sample preparation

S1.2. REAGENT SETUP

General preparation. We recommend that all the glassware be washed with *aqua regia* Including disposable glassware used as reaction vessels for the nanomaterial syntheses. We recommend the use of disposable glass pipettes. Moreover, we do not sonicate or vortex any precursor solution during the nanoparticle preparation; we mildly shake and keep all solution capped before using them at a constant temperature. Every gold precursor solution is always freshly prepared. All ligand exchange reactions were performed under nitrogen purging.

S2. PROCEDURES

S2.1. Synthesis of gold nanoprisms (GNPR) (*60-64*)

Preparation of seed solution ● timing 75 min

1| Place the 20 mL disposable glass vial with the magnetic stirrer on hotplate and set the stir bar to 300 rpm; keep the temperature of the sand bath to 35°C.

2| Once the temperature is stable, CTAC and gold solution were prepared, then 4.7 mL of 0.1 M CTAC (6.4 g in 50 mL DI H_2O) solution was added into the vial followed by 25 μL of 0.05 M $HAuCl_4 \cdot 3H_2O$ (84.9 mg in 5 mL DI H_2O) solution.

3| After 3 minutes, 300 μL of freshly prepared 0.01 M $NaBH_4$ (7.6 mg in 20 mL DI H_2O) was injected under vigorous stirring at 1,000 rpm This seed solution was kept for 2 h at 25 °C to consume the excess of borohydride.

▲CRITICAL STEP: The speed of the stir bar was checked when we used the surfactant;

keeping the mixture in low speed to avoid foam formation. It is essential to set the stirring speed to 1,000 rpm when $NaBH_4$ is added. $NaBH_4$ needs to be fresh prepared, and it should be cold when we added to the reaction (0 °C).

Preparation of first growth solution ● timing 15 min

4| Place 50 mL Erlenmeyer flask on the hotplate with the temperature set to 25 °C, add 1.8 mL of 0.1M CTAC to Erlenmeyer flask then 8 mL of DI H_2O to dilute the surfactant concentration.

5| After 5 minutes, the CTAC solution was uniform; afterward 40 µL of 0.05M $HAuCl_4 \cdot 3H_2O$ solution was added followed by 15 µL of 0.01M NaI (15 mg in 10 mL DI H_2O).

Preparation of second growth solution ● timing 15 min

6| Place a 100 mL Erlenmeyer flask on a hotplate with the temperature set to 25 °C, add 40 mL of 0.05M CTAC solution (prepared in step **2** and diluted to half the concentration of CTAC) to the flask.

7| After 5 minutes the CTAC solution was uniform, and then 500 µL of 0.05M $HAuCl_4 \cdot 3H_2O$ solution was added followed by 300 µL of a 0.01M NaI solution.

Synthesis of gold nanoprism solution ● timing 15 min

8| Before proceeding to the main reaction, the papered seed solution was diluted 10 times in a 0.1M CTAC solution.

9 | 0.1M aqueous solution of ascorbic acid was prepared (176 mg in 10 mL DI H_2O) and added subsequently in portions of 40 µL and 400 µL to first growth (steps 4 & 5) and second growth solution (steps 6 & 7) respectively; both mixtures were mildly shaken with a concomitant color change of the transparent solutions from light brown to yellow, indicating the reduction of Au^{III} to Au^{I}.

10| With a micropipette, 200 µL of diluted seed solution (see step 8) was added to the first growth solution and immediately 3.2 mL of this solution was injected in the second growth solution with a 5 mL plastic syringe.

▲CRITICAL STEP: We recommend not to use a magnetic stir bar for the growth solutions. It is

essential to be fast (less than 3 seconds) when adding the first growth solution to the second growth solution. If the color of the first growth solution changes to the dark pink (only very light pink is suitable), the reaction has already failed, and it is better to prepare the new growth solution and redo steps 4, 5, 9, and 10.

Purification and storage of gold nanoprism solution ● timing 20 h

11| After 5 h, when the reaction is complete, 11 mL of the CTAC stock solution was added and the resulting solution gently shaken. Then, this solution was transferred to a 100 mL glass cylinder flask for 15 h, and then the precipitation was collected (greenish blue color). The gold nanoprisms stuck to the wall of glass cylinder and were washed using 10 mL DI H_2O.

12| Mild agitation by shaking produced a colloidal aqueous solution of the gold nanoprisms, which was transferred to 2 mL volume centrifuge tubes. Centrifugation at 12,000 rpm for 10 minutes was used to isolate the nanoprisms.

13| The supernatant was removed and discarded by disposable pipettes and any remaining product was washed twice and redispersed with DI H_2O.

◆PAUSE POINT: The gold nanoprisms are stable under ambient conditions (room temperature) for several months without changing the shape or quality.

✦COLOR INDEX FOR SOLUTIONS (Step: S):

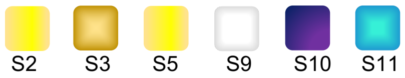

S2.2. SYNTHESIS OF GOLD NANOSTARS (GNS) (*59*)

Full procedure ● timing 6 h

1| The sand bath was set to a temperature of 40 °C, then a larger magnetic stir bar was placed into a 100 mL Erlenmeyer in the sand bath and allowed to temperature equilibrate for 15 min.

2| Once the temperature was reached and constant, 8.4 g of Triton X-100 was added using a 10 mL pipette. The spin rate of stir bar set between 500 – 600 rpm and the heat of sand bath was set to 45 °C.

3| A 1.25mM gold salt solution was prepared in the 20 mL disposable glass vial (9 mg of $HAuCl_4 \cdot 3H_2O$ dissolved in 11.6 mL of DI water). The gold solution was injected to the surfactant solution of Triton X-100 in water in 4 steps over 40 seconds. The spin rate of the stir bar was increased to 900 rpm and the mixture stirred for 1 h at 35 °C to obtain a homogeneous mixture.

4| A 2mM silver salt solution was prepared in a 20 mL disposable glass vial by dissolving 3.4 mg of $AgNO_3$ in 10 mL of DI water. This solution was added to the mixture (step **3**) and the spin rate was kept at 1,000 rpm for 5 minutes to produce a homogenous mixture.

5| In a 10 mL disposable glass vial a 20mM solution of *L*(+)-ascorbic acid in DI water was prepared and 2.5 mL of this solution was added to the mixture rapidly.

6| The mixture (step **5**) was removed from the sand bath and shaken mildly, and it was kept at 25 °C for 5 h undisturbed.

7| The colloidal mixture was diluted 2-times with DI H_2O then was transferred to 2 mL volume centrifuge tubes to concentrate the product via centrifugation at 12,000 rpm for 15 minutes. Then, the supernatant was removed and discarded by disposable pipettes. Any remaining product was washed 3-times and redispersed with DI H_2O.

▲CRITICAL STEP: The temperature should be carefully set up to avoid aggregation of gold solution in the hexagonal columnar phase of the liquid crystal template (Triton X-100). Also, if Triton X-100 is not warm enough it causes a gradient concentration of the surfactant. Triton X-100 is very viscous, so it is recommended that one uses a sufficiently large amount of Triton X-100 for the transfer to the reaction flask. In preparation steps 1 to 4, adding $AgNO_3$ does not produce any obvious color change, but by adding ascorbic acid the mixture progressively turned from yellow to transparent; at first pink, and finally to a greenish blue color that darkens with time.

◆PAUSE POINT: Gold nanostars are stable under ambient conditions (room temperature) for several month without changing the shape or quality.

✦COLOR INDEX FOR SOLUTIONS (Step: S):

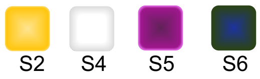

S2.3. SYNTHESIS OF HIGH ASPECT RATIO GOLD NANODISKS (H_{AR}-GND) (*56*)

Full procedure ● timing more than 48h

1| This synthesis follows the procedure (steps 1 – 13) described for the gold nanoprism (section S2.1).

2| The temperature of the sand bath was set to 30 °C, then a sufficiently large magnetic stir bar was placed inside a 100 mL Erlenmeyer flask. Once the temperature equilibrated (ca. 15 min), 50 mL of the gold nanoprism solution were added.

3| Thereafter, 500 µL of 1M HCl and 400 µL of H_2O_2 (10 wt.% in water) were prepared and injected into the nanoprism solution. The spin rate of stir bar was set between 500 and 600 rpm and the mixture stirred for 48 h.

4| The mixture was then transferred to 2 mL volume centrifuge tubes and concentrated by centrifugation at 10,000 rpm for 10 minutes. Then, the supernatant was removed and discarded using disposable glass pipettes. The remaining product was washed 2 times and redispersed in DI H_2O.

▲CRITICAL STEP: Gold nanodisks with varying diameters (undesired large diameter distribution). By carefully controlling the oxidation reaction every 12 h (using TEM) and using exact concentrations of the HCl and H_2O_2 solutions (step 3) gold nanodisks with quasi-identical diameter and thickness can be obtained.

◆PAUSE POINT: Gold nanodisks with smaller diameter are stable under ambient conditions for a week without changing shape or quality when stored at room temperature and at neutral pH.

✦COLOR INDEX FOR SOLUTIONS (Step: S):

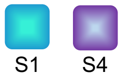

S2.4. SYNTHESIS OF LOW ASPECT RATIO GOLD NANORODS (L_{AR}-GNR) (*65*)

Preparation of seed solution ● timing 90 min

1| Place a 20 mL disposable glass vial with a magnetic stir bar on a hot plate stirrer and set the stir bar speed to 300 rpm; set the temperature of the sand bath at 30 °C.

2| Prepare a gold precursor solution containing 500 µL of 0.01M $HAuCl_4 \cdot 3H_2O$ in DI water.

3| Prepare 100 mL of 0.1M CTAB solution in DI H_2O. Once the temperature of the sand bath and flask has equilibrated (ca. 15 min), 9.5 mL of the CTAB solution was injected to the vial and mixed with gold solution.

4| After 5 minutes, 500 µL of a freshly prepared, ice-cold 0.01M $NaBH_4$ solution (7.6 mg in 10 mL of 0.01M NaOH in DI water solution) was prepared and injected under vigorous stirring at 1,100 rpm. This seed solution was kept for 2 h at 25 °C to consume excess borohydride.

Preparation of growth solution ● timing 30 min

5| In disposable glass vials, stock solutions of 0.1M $AgNO_3$ (17 mg in 10 mL DI H_2O) and 0.1M ascorbic acid (176 mg in 10 mL DI H_2O) were prepared.

6| Five 20 mL precleaned disposable glass vials were used to prepare growth solution: to each vial 8 mL of CTAB, 0.5 mL of $HAuCl_4 \cdot 3H_2O$ and 20 µL of $AgNO_3$ were added, respectively, and the sealed vials (solutions) were gently inverted to homogenize the solutions.

7| To each solution, 50 µL of 1M aqueous HCl and 80 µL of the ascorbic acid solution were introduced, sealed, and gently inverted again.

8| Once these solutions become transparent, 2 mL of seed solution (Steps 1 – 4) was added and vigorously mixed for 30 second.

9| The solutions were kept undisturbed for 16 h at exactly 28 °C, and the nanorods were isolated the next day by centrifugation at 11,000 rpm for 10 minutes. The resulting pellets in the centrifuge tubes were collected and redispersed in vials in DI water using 2 mL volume plastic vials. The colorful supernatant was collected and purified in the same way 2 times to collect as much of the nanorods as possible.

▲CRITICAL STEP: Smaller gold nanorods with varying aspect ratios might be obtained if the nanoparticle seeds in the seed solution are not uniform (size and shape – check by TEM). The HCl concentration of the growth solution remains critical since the reduction potential is pH dependent. By controlling the centrifuge speed, time, and concentration of the HCl solution, gold nanorods with nearly identical aspect ratio can be produced.

✦COLOR INDEX FOR SOLUTIONS (Step: S):

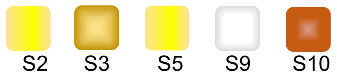

S2.5. SYNTHESIS OF HIGH ASPECT RATIO GOLD NANORODS (HAR-GNR) (*62*)

Preparation of seed solution ● timing 90 min

1| Use steps of 1 to 4 as described above in section 2.5 for the seed solution preparation.

▲CRITICAL STEP: Tuning the pH of the DI H_2O using 1M HCl while the CTAB solution is prepared (pH = 5.5).

Preparation of growth solution ● timing 60 min

2| In two precleaned, disposable glass vials, stock solutions of 0.3M $AgNO_3$ (51 mg in 10 mL DI H_2O) and 0.1M of hydroquinone were prepared, respectively.

3| Five 20 mL disposable glass vials were precleaned for the preparation of the growth solution; to each vial, 8 mL of 0.1M CTAB, 0.5 mL of 0.01M $HAuCl_4 \cdot 3H_2O$ and 100 µL of 0.1M $AgNO_3$ (all aqueous) were added, respectively, and the sealed vials (solutions) were gently inverted.

4| 40 µL of 1M HCl was introduced to each vial, and the sealed vials again gently inverted. These solutions then remained at rest for 30 min.

5| Thereafter, 500 µl of hydroquinone was added to each vial, and the vials gently swirled.

6| Once these solutions become transparent, 2 mL of seed solution was injected to the mixture. The solutions were kept undisturbed for 16 h at exactly 28 °C and were purified the next day. To do so, the mixture was transferred to 2 mL plastic centrifuge vials and centrifuged at 10,000 rpm for 10 minutes. The colorful supernatant was collected and purified using the same method 2 times to collect as much of the nanorods as possible.

▲CRITICAL STEP: The pH of DI H_2O was adjusted to 5.5 for the CTAB solution. To obtain nanorods with high aspect ratio, the weak reducing agent hydroquinone was used instead of ascorbic acid. The exact concentration of the HCl solution is critical for producing nanorods with higher aspect ratio.

◆PAUSE POINT: Under ambient conditions these nanorods are stable for 3 months without changes the shape (aspect ratio) or quality.

✦COLOR INDEX FOR SOLUTIONS (Step: S):

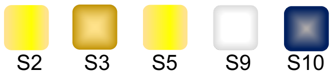

S2.6. LIGAND EXCHANGE

The synthesis and complete spectroscopic characterization of the cholesterol-disulfide (structure shown below) was already described earlier. (*48, 50*)

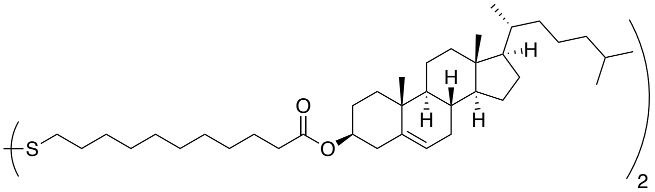

The ligand exchange from CTAB, CTAC, and Triton X-100 to the cholesterol-thiol for the six nanoparticle shapes (H_{AR}-GND, L_{AR}-GND, GNPR, GNS, L_{AR}-GNR, and H_{AR}-GNR followed the same method published previously for the GNP and M_{AR}-GNR. After removing any excess of surfactant by washing the solutions of the nanoshapes twice for CTAB and CTAC and once for Triton X-100 coated particles with a 1:1 ratio of chloroform/DI water, 4 mL of the nanoshape solution in a 10 mL glass vial and 4 mL (1:1 = *v*/*v*) of cholesterol-disulfide were dissolved in chloroform and mixed. Then, adjustment of the pH to basic conditions (pH ~ 10) using a few drops of aqueous 1M NaOH solution provided nanoshapes functionalized by the cholesterol-thiol after stirring overnight. After washing to remove any excess of free ligand, the capped nanoshapes were mixed with the N-LC 5CB and remained well dispersed as shown in the photo below. There was no sign of aggregation or settling at the concentrations investigated, and these dispersions remained stable for at least several days.

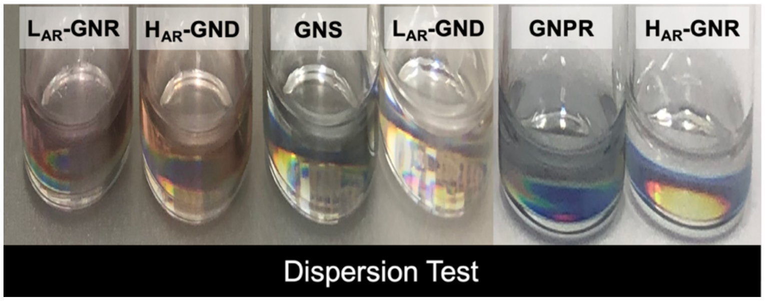

Fig. S1. Dispersions of gold nanoshapes in 5CB. Photographs of vials showing the dispersions of the gold nanoshapes in the induced N*-LC phase formed by 5CB at T = 25 °C.

S3. CHARACTERIZATION

S3.1. TRANSMISSION ELECTRON MICROSCOPY

S3.1.1 Additional TEM images

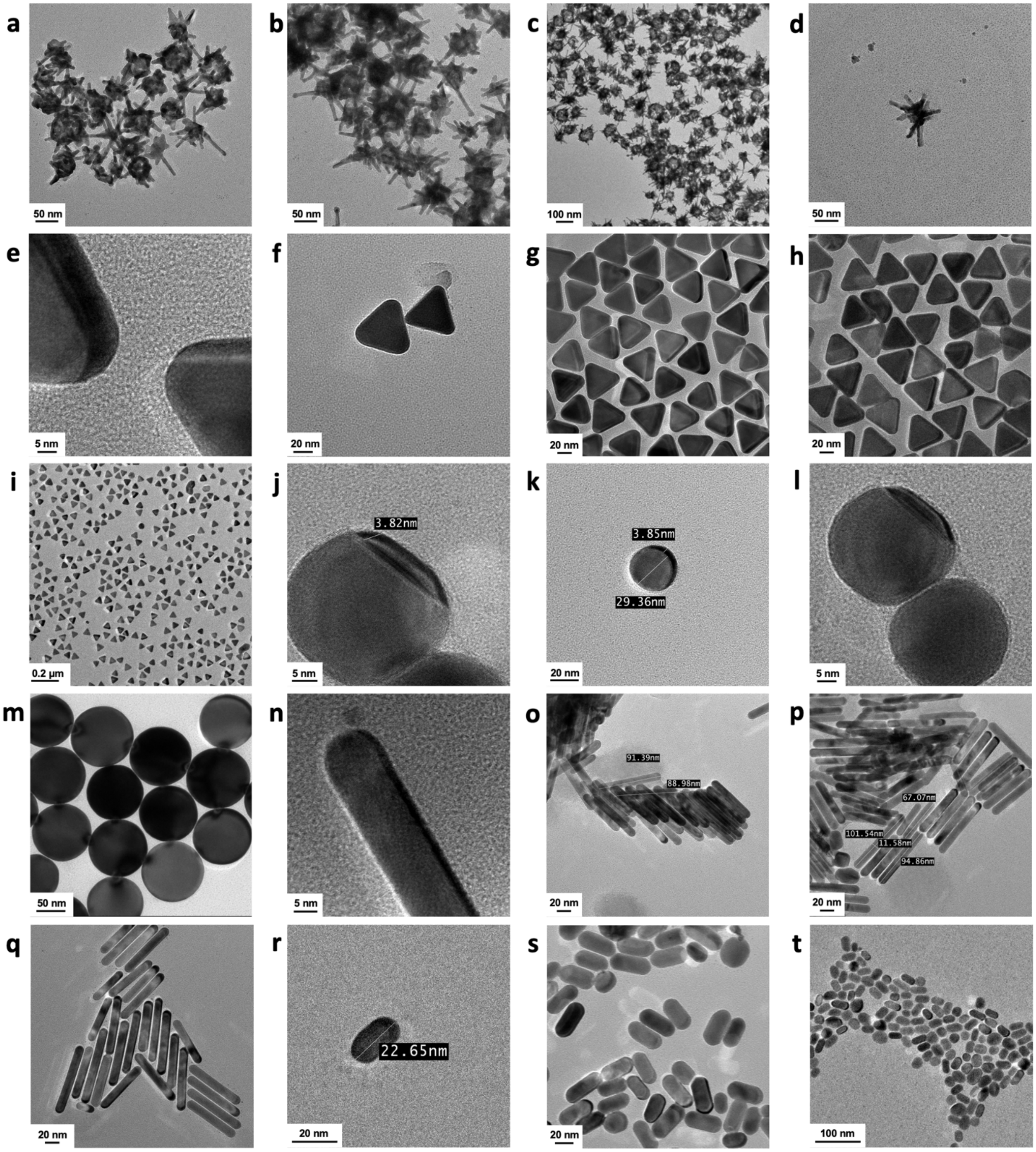

Fig. S2. Additional transmission electron microscopy (TEM) images of the various gold nanoshapes. a – **d** Triton X-100-capped GNS, **e** – **i** CTAB/CTAC-capped GNPR, **j** – **l** CTAB/CTAC-capped H_{AR}-GND, **m** CTAB/CTAC-capped L_{AR}-GND, **n** – **q** CTAB-capped H_{AR}-GNR, and **r** – **t** CTAB-capped L_{AR}-GNR.

S3.1.2 Size distributions for the various gold nanoshapes

The average size distributions were obtained by ImageJ® (*66*); table colors match the absorption band of the nanoshape in the visible portion of the EM spectrum.

Tables S1 – S6. Size and size distribution of the cholesterol-thiol-capped gold nanoshapes. Size distributions derived from the analysis of the TEM images using the image analysis software ImageJ®. Average aspect ratio, *AR*, shown below is for the gold nanoshape cores.

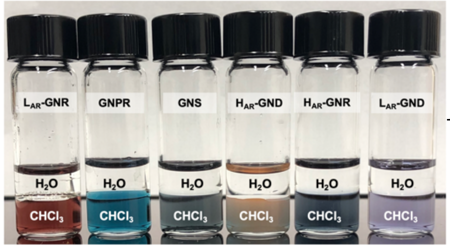

**Table S1.** GNS	Size (nm)
Core	60.0 ± 6.5
Spikes	69.0 ± 8.4
Average of all (core + spikes)	178.0 ± 12.8

**Table S2.** GNPR (*AR* ~ 4)	Size (nm)
Length of sides	49.8 ± 2.7
Height	12.5 ± 1.0

**Table S3.** H_{AR}-GNR (*AR* ~ 8.5)	Size (nm)
Length	87.0 ± 12.5
Diameter	10.2 ± 1.0

**Table S4.** L_{AR}-GNR (*AR* ~ 1.7)	Size (nm)
Length	25.0 ± 1.7
Diameter	15.2 ± 1.5

**Table S5.** H_{AR}-GND (*AR* ~ 6.1)	Size (nm)
Diameter	45.0 ± 3.2
Height	7.4 ± 1.5

**Table S6.** L_{AR}-GND (*AR* ~ 3.7)	Size (nm)
Diameter	79.0 ± 2.0
Height	21.6 ± 0.8

The dimensions of the size distributions of previously reported cholesterol-thiol functionalized gold nanoshapes are as follows: GNP_5: diameter = (5.5 ± 1.2) nm; GNP_{10}: diameter = (10.0 ± 2.7) nm; M_{AR}-GNR: $l \times w$ = (43.0 ± 3.3) × (10 ± 1.3) nm (*AR* = 4.3). For comparison: AR_{5CB} ~ 4.0.

S3.2. *VIS*-NIR SPECTROSCOPY

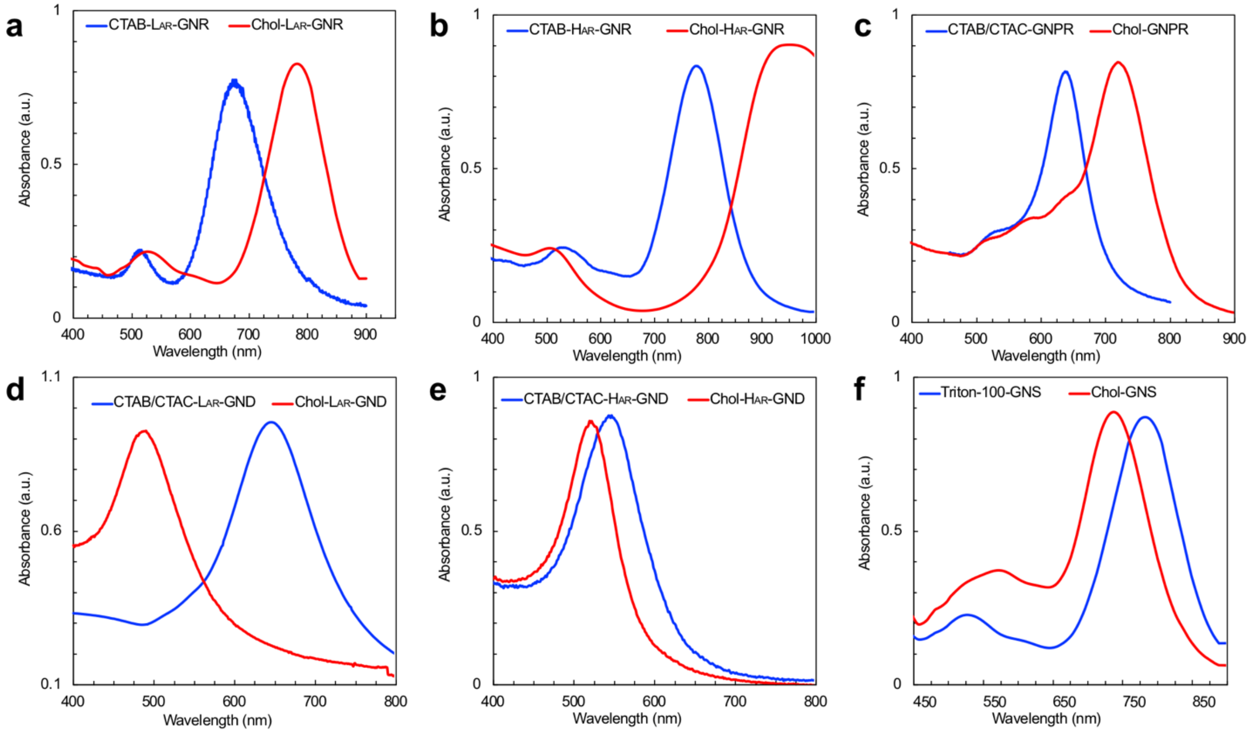

Fig. S3. UV-*vis*-NIR spectra of the precursor, surfactant-capped nanoshapes in H_2O and for all chiral cholesterol-capped particles: **a** CTAB-capped (blue spectrum) and cholesterol-thiol-capped L_{AR}-GNR (red spectrum), **b** CTAB-capped (blue spectrum) and cholesterol-thiol-capped H_{AR}-GNR (red spectrum), **c** CTAB/CTAC-capped (blue spectrum) and cholesterol-thiol-capped GNPR (red spectrum), **d** CTAB/CTAC-capped (blue spectrum) and cholesterol-thiol-capped H_{AR}-GND (red spectrum), **e** CTAB/CTAC-capped (blue spectrum) and cholesterol-thiol-capped L_{AR}-GND (red spectrum), and **f** Triton X-100-capped (blue spectrum) and cholesterol-thiol-capped GNS (red spectrum). Ligand exchange is indicated by transfer of the nanoshapes to the organic phase as well as shifts of the various SPR bands; hypsochromic for GNRs and GNPR and bathochromic for GNDs and GNS.

S3.3. Circular dichroism (CD) spectropolarimetry

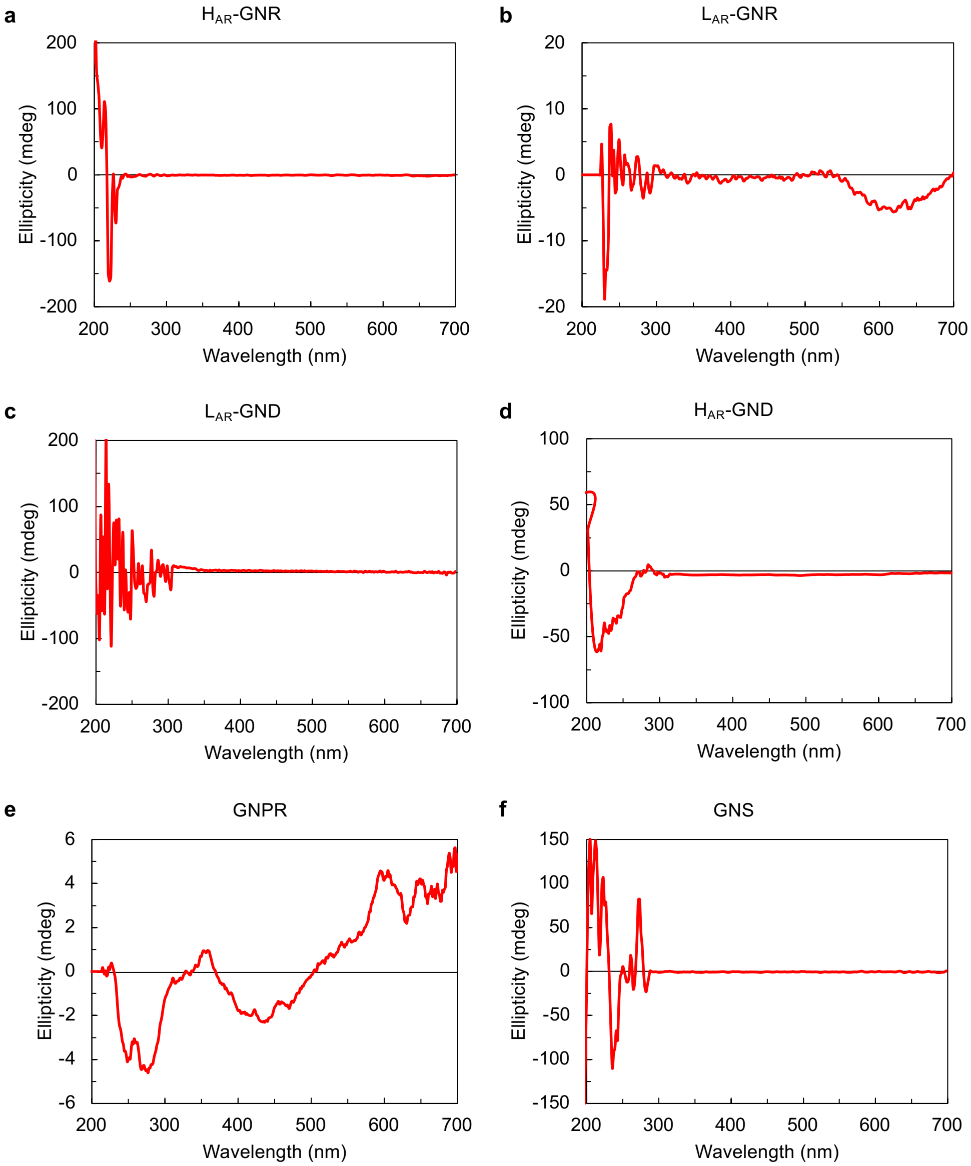

Fig. S4. Solution CD spectra. Solution circular dichroism (CD) spectra of the different cholesterol-thiol capped gold nanoshapes recorded in *n*-hexane: **a** HAR-GNR, **b** LAR-GNR, **c** LAR-GND, **d** HAR-GND, **e** GNPR, and **f** GNS.

S3.4. THERMO-GRAVIMETRIC ANALYSIS (TGA)

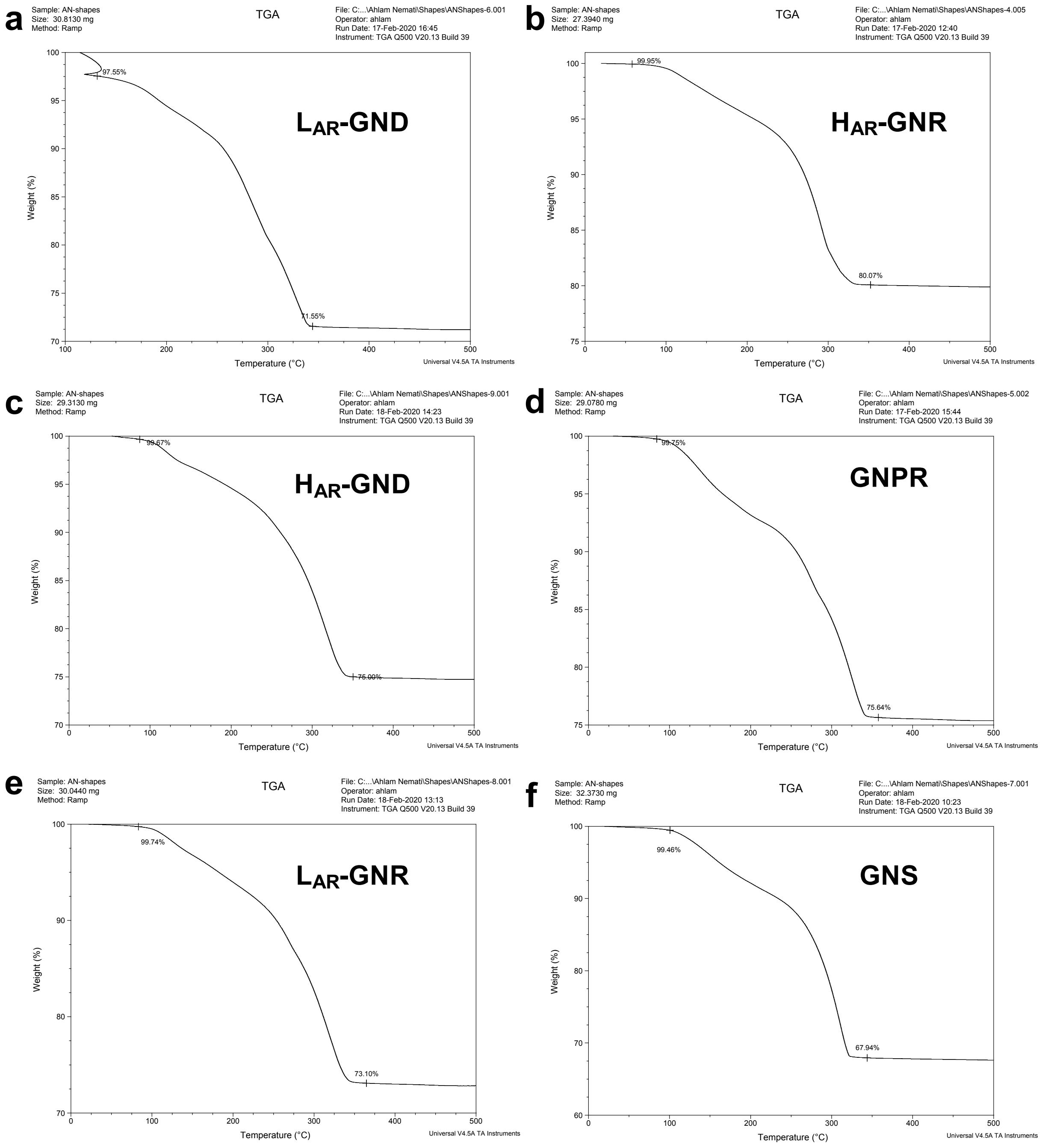

Fig. S5. Determination of ligand coverage (wt.%). Thermogravimetric analysis (TGA) plots for the cholesterol-thiol-capped nanoshape samples: **a** cholesterol-thiol-capped L_{AR}-GND (using a 10.61 mg sample; weight loss = 28.4%), **b** cholesterol-thiol-capped H_{AR}-GNR (using a 27.39 mg sample; weight loss = 19.9%), **c** cholesterol-thiol-capped H_{AR}-GND (using a 29.31 mg sample; weight loss = 25%), **d** cholesterol-thiol-capped GNPR (using a 29.07 mg sample; weight loss = 24.3%), **e** cholesterol-thiol-capped L_{AR}-GNR) (using a 32.04 mg sample; weight loss = 20.09%), and **f** cholesterol-thiol-capped GNS (using a 32.37 mg sample; weight loss = 32.1%).

S3.5. COMPARISON BETWEEN TGA AND CALCULATED WEIGHT LOSS DATA

Table S7. Comparison of TGA and calculated ligand coverage. Measured weight loss determined by thermogravimetric analysis in comparison to the calculated ligand coverage (wt.%) of the cholesterol-thiol ligands on the different nanoshapes.

Nanoshape	wt.%$_{\text{Ligand}}$ (calcd.)[a]	wt.%$_{\text{Ligand}}$ (TGA)[b]
**L$_{\text{AR}}$-GNR**	22 ± 5	20.9
**H$_{\text{AR}}$-GNR**	23 ± 4	19.9
**L$_{\text{AR}}$-GND**	29 ± 2	28.4
**H$_{\text{AR}}$-GND**	25 ± 2	25.0
**GNPR**	27 ± 4	24.3
**GNS**	30 ± 6	32.1

[a] The calculated wt.%$_{\text{Ligand}}$ range is taking the size distribution of the nanoshapes (obtained by TEM image analysis) and therefore the calculated surface area ranges into account (see equations below). [b] The ligand coverage of the synthesized and purified nanoshapes was obtained by thermogravimetric analysis (TGA); pure gold was obtained as a residue after heating above 500 °C.

Equations used:

Number of gold atoms on the surface: $N_{Au} = \frac{\mathrm{A}}{A_{Au}}$

Number of ligands on the surface: $N_{\text{Ligands}} = \frac{A}{Surface\ area\ per\ thiol}$

M_{w} of Au atoms in nanoshapes (g mol^{-1}): $M_{\text{w}}(\text{Au in Nanophapes}) = N_{\text{Au}} \times M_{\text{w}}(\text{Au})$

Weight percent of ligands in one nanoshape: $\text{wt.}\,\%_{Ligand} = \frac{m\ (\text{ligands})}{m\ (\text{ligand–capped nanoshape})} \times 100\%$

Surface area:

L$_{\text{AR}}$-GND and H$_{\text{AR}}$-GND: $A = 2\pi rh + 2\pi r^2$

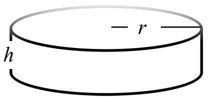

GNPR: $A = bh + H(s_1 + s_2 + s_3);\ with\ b = s_1 = s_2 = s_3 \rightarrow A = bh + 3bH$

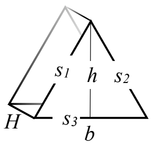

GNS: Surface area of spherical shape + surface area of thorns (cone shape assumed) – surface area of the base of thorns = circular area); surface area of a cone: $A = \pi r\ (r + \sqrt{h^2 + r^2})$

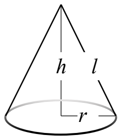

L$_{\text{AR}}$-GNR and H$_{\text{AR}}$-GNR: see calculations reported previously. (*40*)

S4. MEASUREMENT OF THE HELICAL PITCH IN THE INDUCED N*-LC PHASE

S4.1. POLARIZED OPTICAL MICROSCOPY – FREE SURFACE

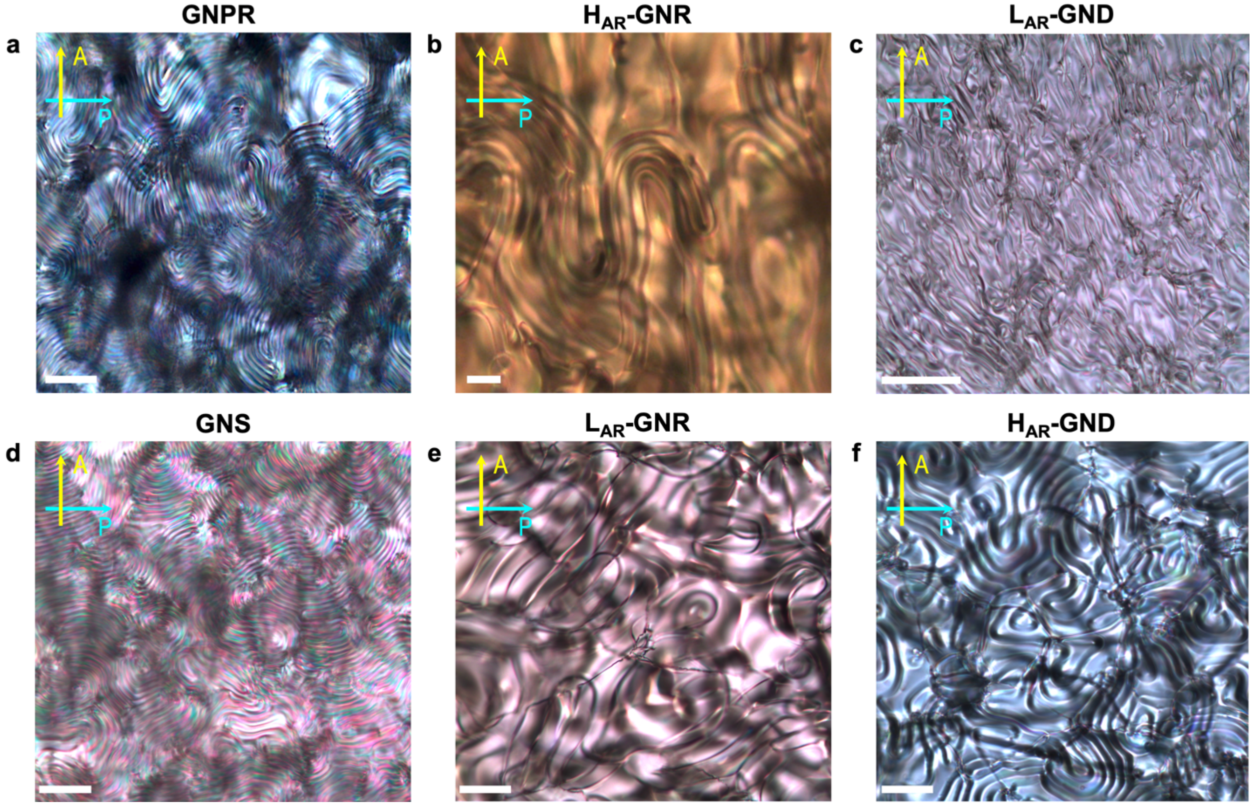

Fig. S6. Pitch measurements – free surface. Polarized optical photomicrographs of the induced N*-LC textures used for helical pitch measurements using the free surface method (crossed polarizers): **a** – **f** 5CB doped with 0.5 wt.% of the different nanoshapes (scale bars = 50 μm).

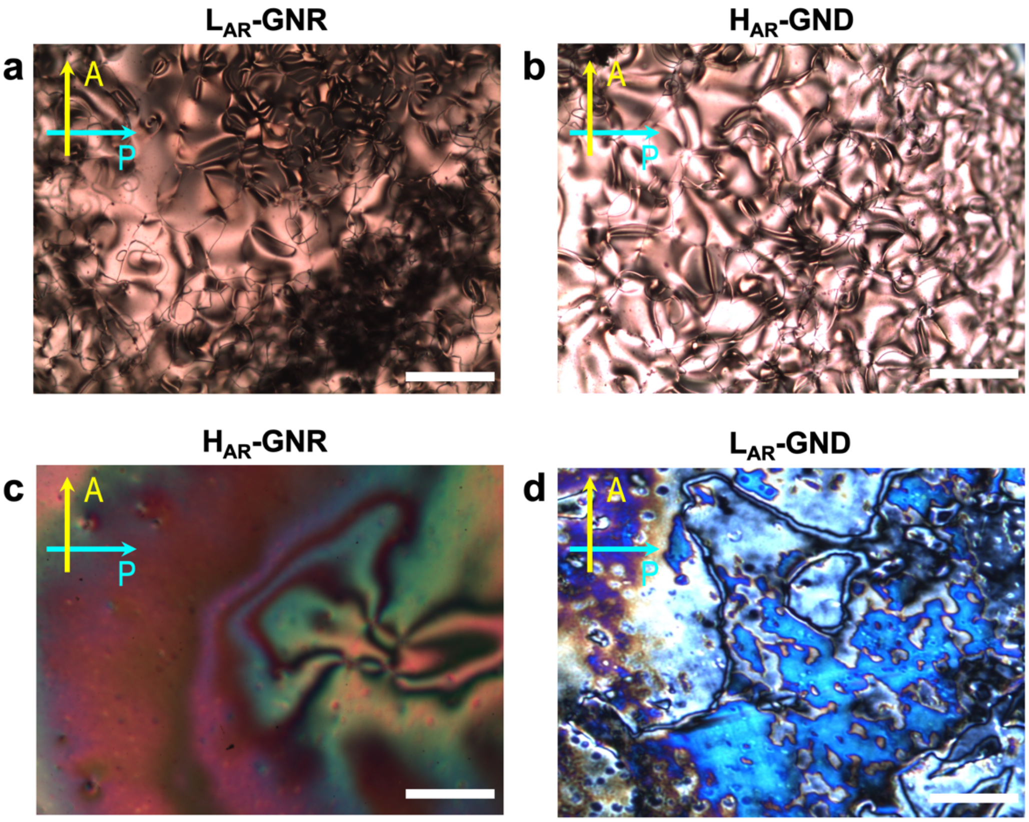

Fig. S7. Pitch measurements – free surface. Polarized optical photomicrographs obtained on cooling from the isotropic liquid phase at 20 °C (crossed polarizer P and analyzer A) for helical pitch measurements using the free surface method: **a** – **d** 5CB doped with 0.4 wt.% of different nanoshapes (scale bars = 100 μm). Doping less than 0.5 wt.% of the cholesterol-capped nanoshape dopants results in large helical pitch values that cannot be measured with any of the pitch measurement methods described here. Onsets of fingerprint textures can be seen in **a** and **b** for L_{AR}-GNR and H_{AR}-GND, but images **c** and **d** show textures typical for achiral nematic LC phases (*Schlieren* textures with disclination lines, *i.e.,* +1 and –1 defects) for H_{AR}-GNR and L_{AR}-GND.

S4.2. Polarized optical microscopy – Grandjean-Cano lens method

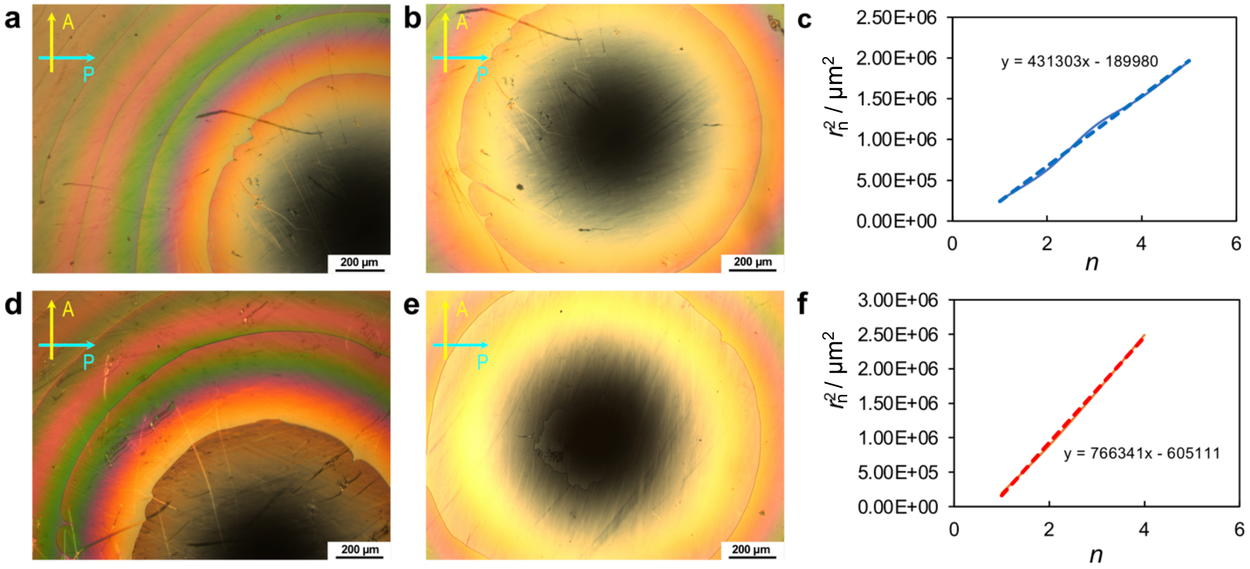

Fig. S8. Pitch measurements – Grandjean-Cano lens method. Polarized optical photomicrographs from Grandjean-Cano lens experiments for determining *p* using a plano-convex lens and a flat glass substrate both treated with rubbed polyimide to induce planar anchoring; photomicrographs (crossed polarizer P and analyzer A) were taken at 25 °C on cooling from the isotropic liquid phase; 5CB doped with 0.5 wt.% of: **a**, **b** cholesterol-thiol-capped GNPR; **c** shows the plot of the square of radius (r_n^2) *vs.* number of Cano lines (n) – details are given below the figure caption, and **d**, **e** cholesterol-thiol-capped GNS; **d** shows the plot of the square of radius (r_n^2) *vs.* number of Cano lines (n). The helical pitch for the GNPR is p = 8.4 µm and for the GNS p = 14.8 µm (all scale bars = 200 µm).

Details for Grandjean-Cano method using a planoconvex lens (*50*):

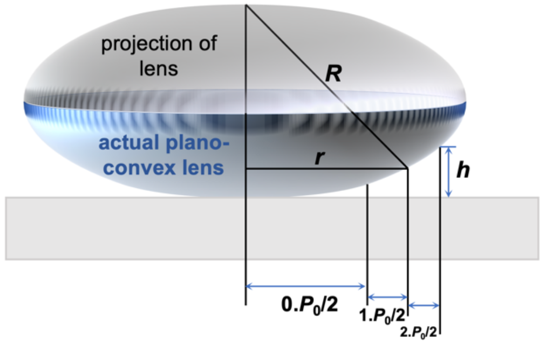

$$h_n = R - (R^2 - r_n^2)^{0.5} \approx R - R\left(1 - \frac{1}{2}\left[\frac{r_n}{R}\right]^2\right) = \frac{r_n^2}{2R}\ (when\ r \ll R)$$

From the linear wedge case: $r_n^2 = (P_0 R)_n + \frac{1}{2} P_0 R$.

Thus, $h_n = \frac{1}{2} P_0 n + \frac{1}{4} P_0$ and $\frac{r_n^2}{2R} = \frac{1}{2} P_0 n + \frac{1}{4} P_0$.

We plot r_n^2 *vs.* n, where: $slope = P_0 R$; $pitch = \frac{Slope}{R}$.

S4.3. PITCH MEASUREMENT IN N*-LC PHASE (HOMEOTROPIC CELLS) OF GNPR & GNS

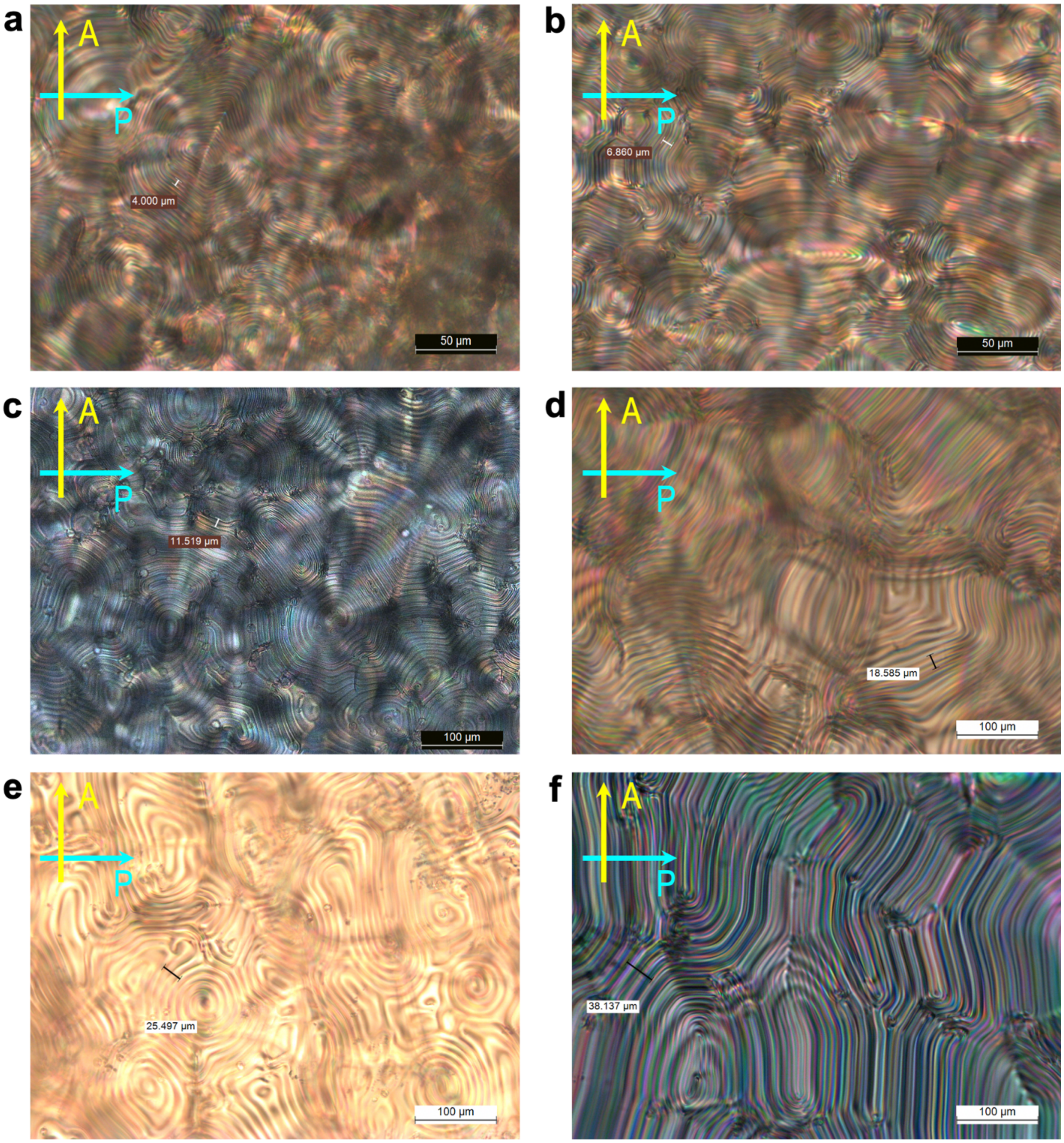

Fig. S9. **Pitch measurements – homeotropic anchoring.** Polarized optical photomicrographs (crossed polarizer P and analyzer A) obtained on cooling from the isotropic liquid phase at 20 °C of the N*-LC phase induced by: **a** 0.7 wt.% GNPR showing a fingerprint texture with p ~ 4 µm, **b** 0.6 wt.% GNPR showing a fingerprint texture with p ~ 6.8 µm, **c** 0.4 wt.% GNPR showing a fingerprint texture with p ~ 11 µm, **d** 0.3 wt.% GNPR showing a fingerprint texture with p ~ 18.5 µm, **e** 0.2 wt.% GNPR showing a fingerprint texture with p ~ 25 µm, and **f** 0.1 wt.% GNPR showing a fingerprint texture with p ~ 38 µm.

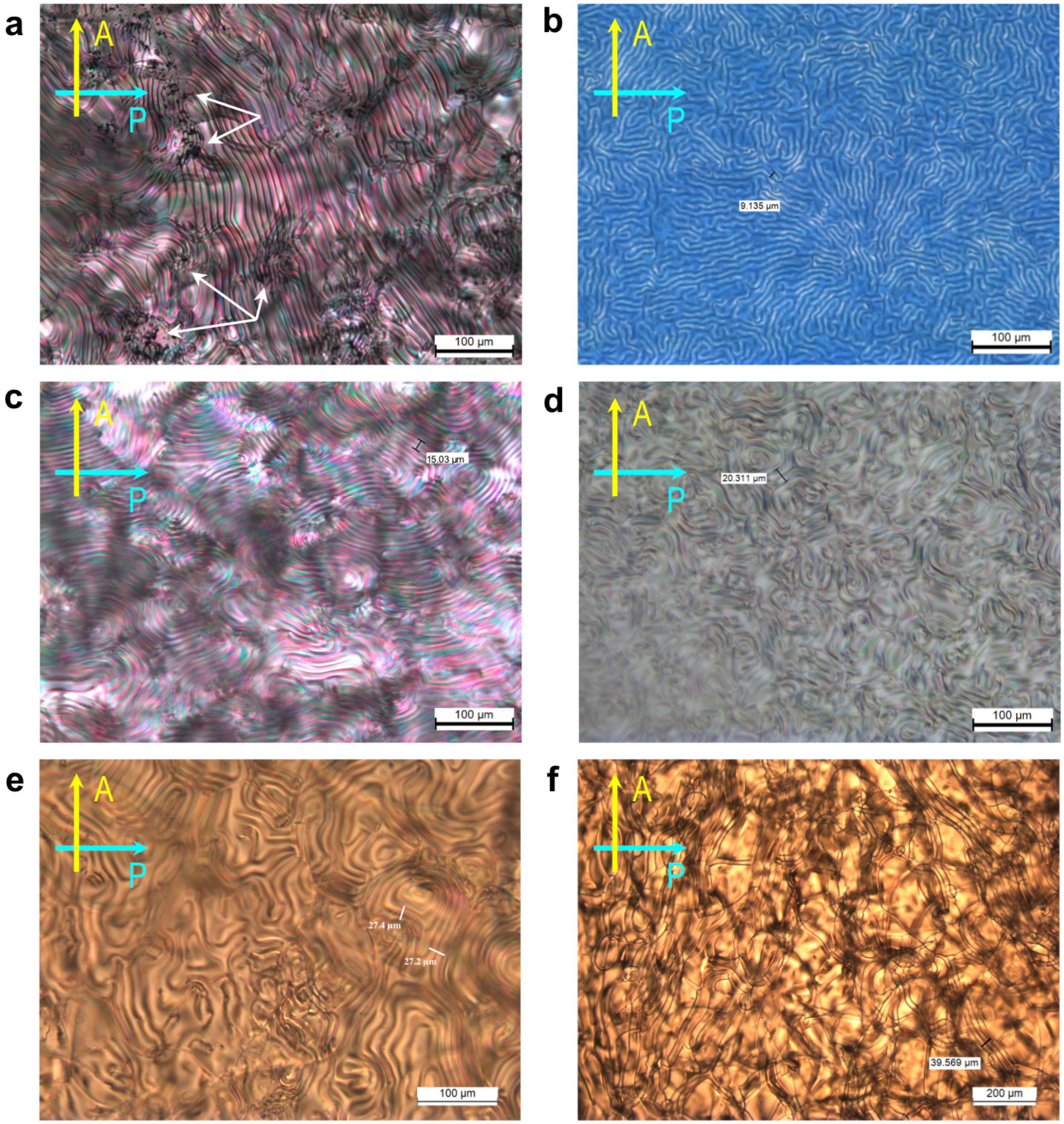

Fig. S10. Pitch measurements – homeotropic anchoring. Polarized optical photomicrographs (crossed polarizer P and analyzer A) obtained on cooling from the isotropic liquid phase at 20 °C of the N*-LC phase induced by: **a** 0.7 wt.% GNS showing a fingerprint texture but with some GNS aggregation (white arrows – pitch is measurable but due to the already visible aggregation likely not fully accurate – undervalued), **b** 0.6 wt.% GNS showing a fingerprint texture with $p \sim 9$ µm, **c** 0.5 wt.% GNS showing a fingerprint texture with $p \sim 15$ µm, **d** 0.4 wt.% GNS showing a fingerprint texture with $p \sim 20$ µm, **e** 0.3 wt.% GNS showing a fingerprint texture with $p \sim 27$ µm, and **f** 0.2 wt.% GNS showing a fingerprint texture with $p \sim 39$ µm.

S5. THIN FILM ICD DATA

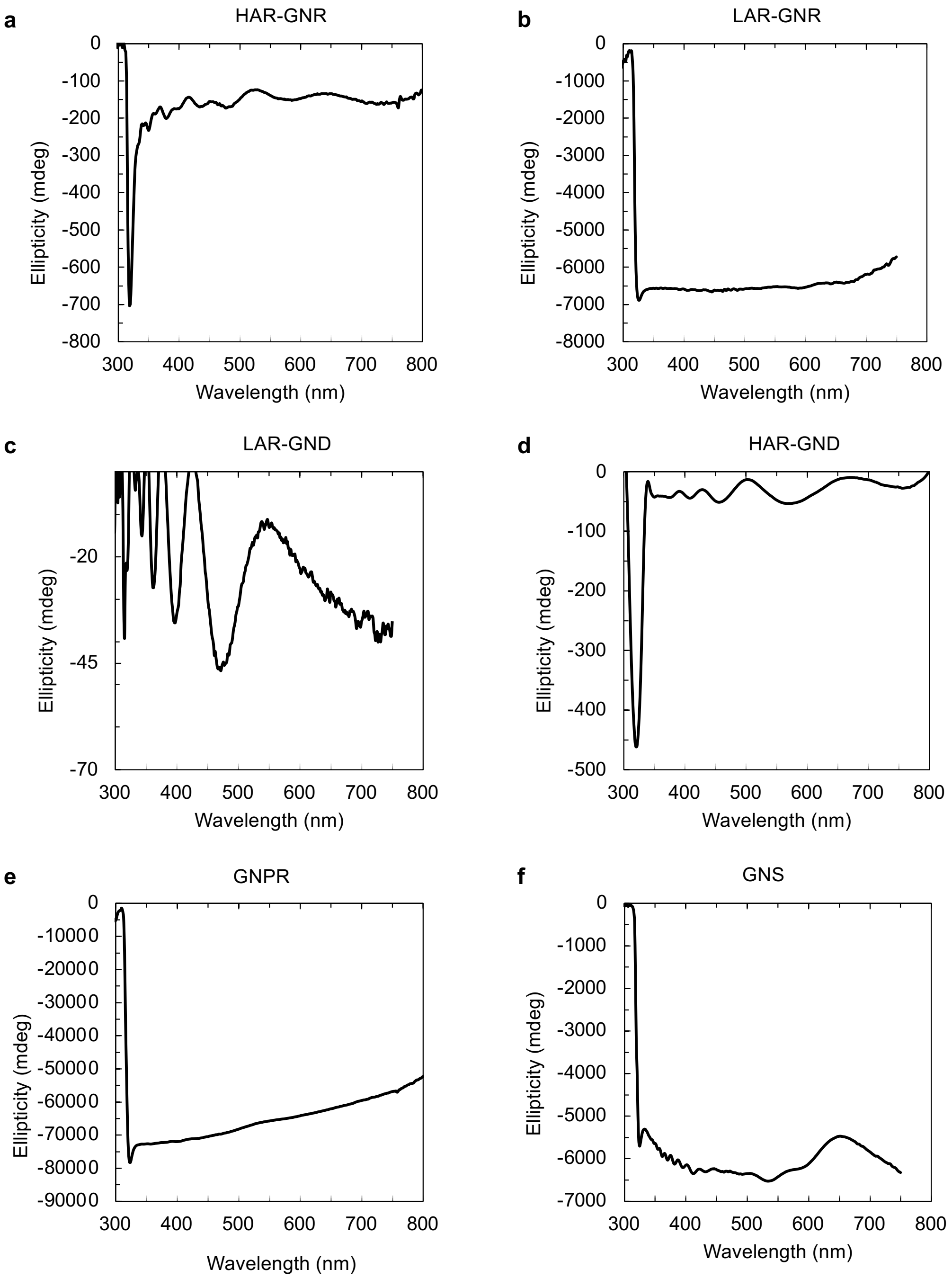

Fig. S11. Thin film induced circular dichroism (ICD). ICD spectra of dispersions of 0.5 wt.% of the cholesterol-thiol-capped nanoshapes in 5CB sandwiched between two quartz substrates at 25 °C. **a** H_{AR}-GNR, **b** L_{AR}-GNR, **c** L_{AR}-GND, **d** H_{AR}-GND, **e** GNPR, **f** GNS (all cell gaps = 20 μm).

S6. ANOMALOUS TRENDS IN PLOTS OF p^{-1} *vs.* NANOSHAPE CONCENTRATION

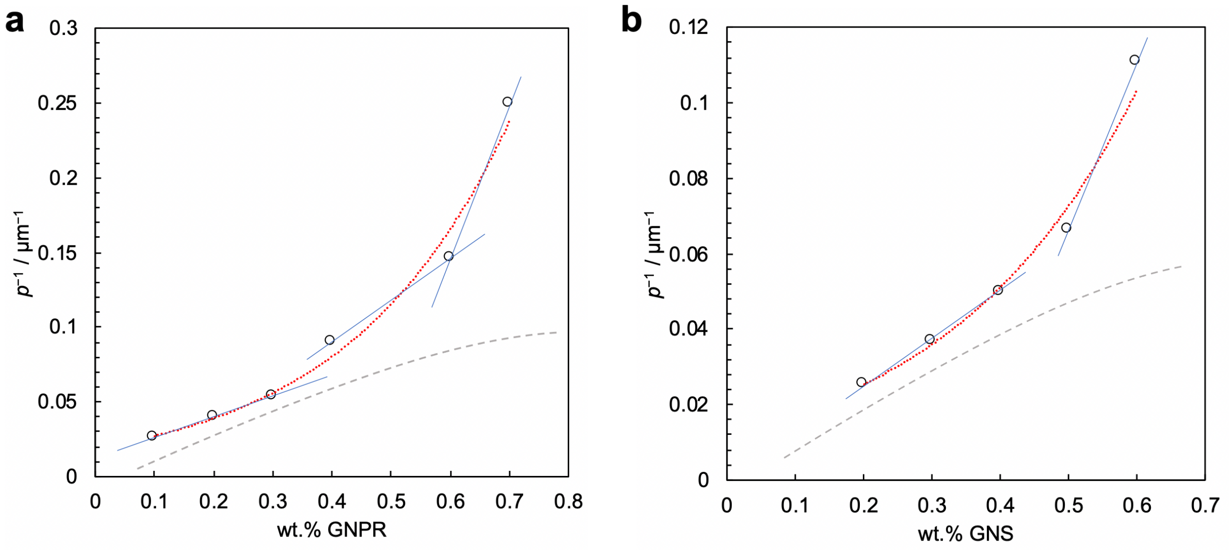

Fig. S12. Plots of p^{-1} *vs.* concentration. Representative plots of the inverse pitch (p^{-1}) *vs.* the concentration of **a** the GNPR and **b** the GNS. The dotted red and the solid blue lines indicate the logarithmic and steadily changing slope of the data points, respectively (data taken from figs. S9 and S10). The hypothetical dashed grey lines show the common trends of the inverse pitch *vs.* the concentration of a typical organic molecule-based chiral additive (or dopants), where the p^{-1} linearly increases with the concentration and then saturates (plateaus) once a certain pitch is reached (*i.e.*, the pitch saturates). This behavior is normally limited to the lower chiral additive concentration regime as higher concentrations frequently lead to either phase separation of the chiral additive or to the induction of other chiral phases (*e.g.*, blue phases; (*68*)). As for the previously reported GNRs (*40*), the experimentally observed increasing slope with increasing nanoshape concentration reveals a cooperativity exerted by the chiral ligand-capped gold nanoshapes, now helically distorted within the induced N*-LC matrix that further contributes to the observed amplification of the chirality transfer efficacy.

S7. CALCULATIONS

S7.1. CALCULATION OF THE PARTICLE-PARTICLE DISTANCE

$$D_{\mathrm{P-P}} = \sqrt[3]{\frac{2 \times Mw\ of\ 5CB \times Number\ of\ 5CB\ around\ nanoshapes}{N_A \times \rho_{5CB}}} - av.\ size_{nanoshapes}.$$

The number of 5CB molecules around each nanoshape assumed the dimensions provided later in Table S8. For more details, see references (*40*, *50*, *69*).

S7.2. CALCULATION OF CHIRALITY INDICES

Calculations of chirality indices G_{oa}^{a} and $G_{oa,max}^{a}$.

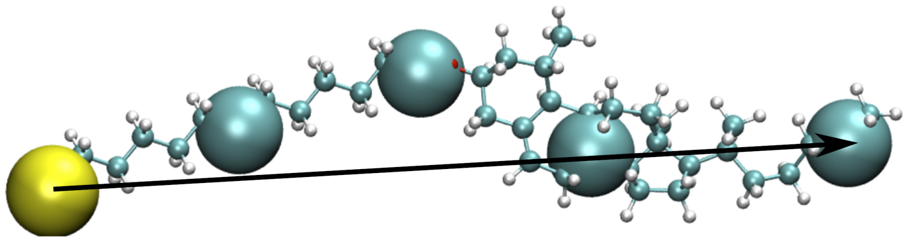

Fig. S13. Coarse-grained (CG) model used for $|G_{oa}^{a}|$ and newly calculated $|G_{oa,max}^{a}|$ calculation. Also shown is the "main axis" vector from the first bead, placed on the S atom, to the last one, positioned in the middle of the terminal C6 chain.

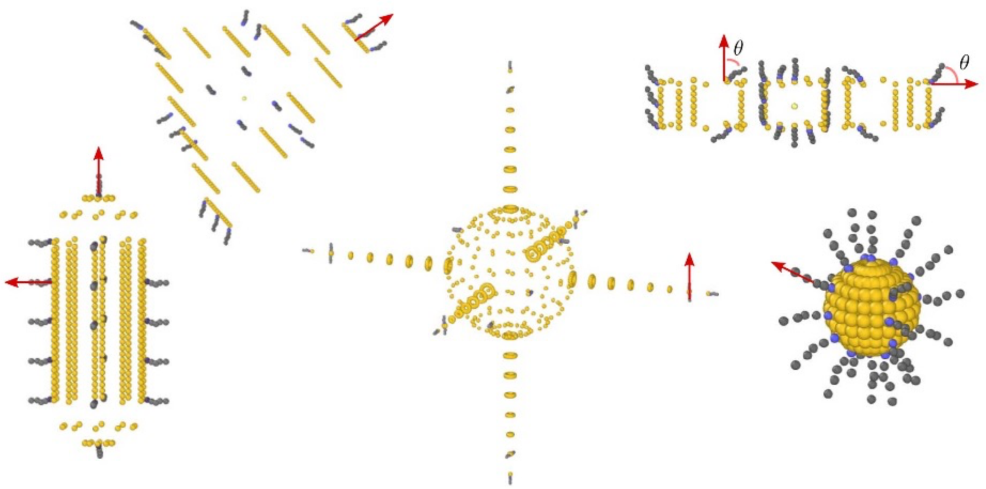

Fig. S14. Computational snapshots of CG-capped nanoshapes. Examples of the nanoshapes decorated with CG ligands as used in chirality computations; from left-to-right: L_{AR}-GNR, GNPR, GNS, GND, GNP. We also show the angle θ used in the calculations.

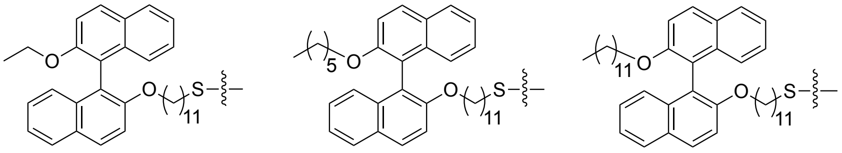

(*S*)- and (*R*)-**1** (*S*)- and (*R*)-**2** (*S*)- and (*R*)-**3**

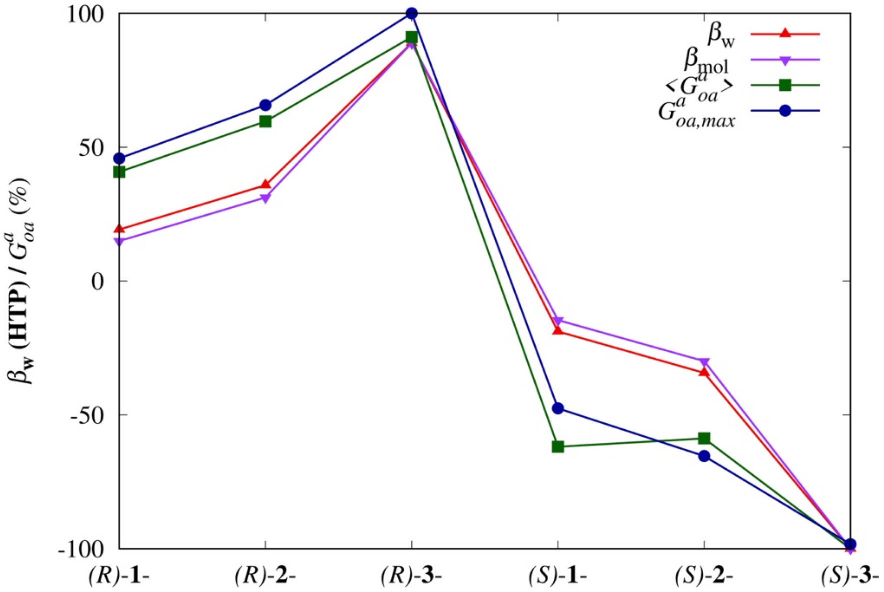

Fig. S15. Comparison between G^a_{oa} and $G^a_{oa,max}$. Trends of previous G^a_{oa} and newly calculated $G^a_{oa,max}$ for MAR-GNRs capped with axially chiral binaphthyl-thiol derivatives (*R*)- or (*S*)-**1** – (*R*)- or (*S*)-**3** (shown above the plot) in comparison to β_w and β_{mol} – using data from and ligands specified in reference (*69*).

S7.3. Isoperimetric ratios

S7.3.1 2-D Isoperimetric ratios

The values of IPR_{2D} and IPR_{3D} are summarized below in Tables S10 and S11 and the differences to the *IPR* (2- and 3-D) of 5CB compared against the β_{mol} data shown in Fig. 4 (lowest difference set to 100%; highest difference to 0%). Calculations of IPR_{2D} and IPR_{3D} values took the energy-minimized length and shape of the cholesterol ligands (forming a monolayer capping each nanoshape) into account. For modified values of the *AR*, see Table 1 in the main manuscript.

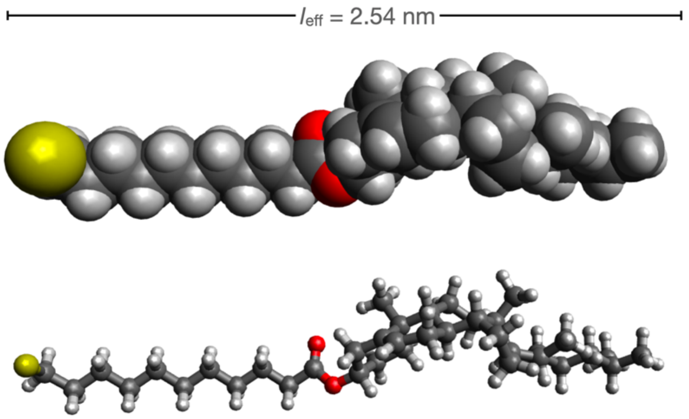

Fig. S16. Energy-minimized 3-D models. 3-D models (ball and stick as well as space-filling) of the energy-minimized shape and effective length (l_{eff}) of the cholesterol-thiol ligand.

Table S8. Calculated Δ(*IPR_{2D}*) values. *IPR_{2D}* of the nanoshapes in comparison to 5CB and other, common N-LCs.

**Nanoshape or N-LC**	Isoperimetric ratio of the related 2D shape	
**5CB**	23.4[a]	
**common N-LCs**	22.0 – 33.5[b]	
		$\Delta\left(\frac{L^2}{A}\right)$ with respect to 5CB
**GNP_5** and **GNP_{10}**	≈13.5[c]	9.9
**L_{AR}-GNR**	14.0[d]	9.4
**M_{AR}-GNR**	24.0[d]	0.6
**H_{AR}-GNR**	36.5 – 44.0[d]	13.1 – 20.6
**L_{AR}-GND** and **H_{AR}-GND**	12.6	10.8
**GNPR**	20.8	2.6
**GNS (average 6 spikes)**	18.7[e]	4.7

[a] Assuming an ellipsoidal shape with an aspect ratio of *AR* ~ 4 (l = 1.8 nm, w_{eff} = 0.45 nm (*70*)) calculated for 5CB (energy-minimized) conformation in the nematic phase with free rotation about the long molecular axis. [b] Assuming an ellipsoidal shape with aspect ratios ranging from *AR* ~ 3 to *AR* ~ 6 calculated for (energy-minimized) conformations in the nematic phase with free rotation about the long axis. [c] Depending on the polyhedral shape that can include a range of Platonic and Archimedean solids (*71*), the most energetically favored truncated octahedron was used (*72*). [d] Based on the average dimensions (length and diameter) of the GNR. [e] Reflects experimental evidence that the number of thorns is on average 6 – 8, but the average number of projected spikes for the 2D projection is about six. Furthermore, their individual length varies, which cannot be precisely included in the calculation. Thus, the average length was used.

S7.3.2 3-D Isoperimetric ratios

Table S9. Calculated Δ(IPR_{3D}) values. IPR_{3D} of the nanoshapes in comparison to 5CB and other, common N-LCs.

Nanoshape or N-LC	Isoperimetric ratio of the related 3D shape	
**5CB**	233.6[a]	
**common N-LCs**	~ 200 – 350[b]	
		$\Delta\left(\frac{A^3}{V^2}\right)$ with respect to 5CB
**GNP_5** and **GNP_{10}**	150.1[c]	83.5
**L_{AR}-GNR**	283.9[d]	50.3
**M_{AR}-GNR**	308.1[d]	74.5
**H_{AR}-GNR**	395.3[d]	161.7
**L_{AR}-GND**	189.8	43.8
**H_{AR}-GND**	241.3	7.7
**GNPR**	477.3	243.7
**GNS (stars with 6 to 8 spikes)**	400.59 – 484.34[e]	250.7 – 167.0

[a–d] See footnotes below table S8. [e] Average value for stars with about 6 to 8 spikes was used, though their individual length varied; the assumed centroid is the truncated octahedral body used as seed for the GNS synthesis.

Table S10. Equations used to calculate IPR_{3D}. Basic equations used to calculate the 3-D isoperimetric ratios.

Nanoshape	Surface Area (A)	Volume (V)
Sphere	$4\pi r^2$	${}^4/_3\,\pi r^3$
Cylinder	$2\pi rh + 2\pi r^2$	$\pi r^2 h$
Truncated Octahedron	$(6 + 12\sqrt{3})a^2$	$8\sqrt{2}a^3$
Triangular Prism[a]	$\sqrt{3}a^2/_2 + 3ah$	${}^3/_4\,Ah$
Hexagonal Pyramid	$\frac{3\sqrt{3}}{2}a^2 + 3a\sqrt{h^2 + \frac{3a^2}{4}}$	$\frac{3\sqrt{3}}{2}a^2 h$
Ellipsoid[a]	$4\pi(\frac{(ab)^{1.6} + (ac)^{1.6} + (cb)^{1.6}}{3})^{1/1.6}$	$\frac{4}{3}\pi abc$

[a] 5CB has an effective width (diameter) of w_{eff} (d_{eff}) = 0.45 nm and length of l = 1.80 nm, giving it an aspect ratio of 4.0. The stated equation is, of course, an approximate value of an ellipsoid's surface area. [b] A truncated cuboctahedron has been used as the 3-D shape for the GNP as this is reported as the equilibrium morphology for *FCC* (face-centered cubic) gold nanoparticles with a diameter > 3 nm (*63*).

The calculated values for the IPR_{3D} are given below:

GNS (6 thorns):

$$\frac{\{(6+12\sqrt{3})+6[\frac{3\sqrt{3}}{2}+3\sqrt{1+\frac{3}{4}}]\}^3}{(8\sqrt{2}+\frac{8\sqrt{3}}{2})^2}=400.59$$

GNS (8 thorns):

$$\frac{\{(6+12\sqrt{3})+8[\frac{3\sqrt{3}}{2}+3\sqrt{1+\frac{3}{4}}]\}^3}{(8\sqrt{2}+\frac{24\sqrt{3}}{2})^2}=484.34$$

GNPR:

$$\frac{[\frac{\sqrt{3}}{2}+3(3.98)]^3}{(\frac{1}{2}3.98)^2}=477.34$$

L_{AR}-GND:

$$\frac{[2\pi(3.66)+2\pi]^3}{(3.66\pi)^2}=189.81$$

GNP_5 and GNP_{10}:

$$\frac{[(6+12\sqrt{3})^2]^3}{(8\sqrt{2})^2}=150.12$$

H_{AR}-GNR:

$$\frac{[2\pi(8.53)+6\pi]^3}{(8.53\pi+\frac{4}{3}\pi)^2}=395.31$$

M_{AR}-GNR:

$$\frac{[2\pi(4.3)+6\pi]^3}{(4.3\pi+\frac{4}{3}\pi)^2}=308.09$$

L_{AR}-GNR:

$$\frac{[2\pi(1.64)+6\pi]^3}{(1.64\pi+\frac{4}{3}\pi)^2}=283.96$$

H_{AR}-GND:

$$\frac{[2\pi(6.08)+2\pi]^3}{(6.08\pi)^2}=241.29$$

S7.4. $\left|G_{oa,max}^{a}S^{XN}\right|$ APPLIED TO PREVIOUS DATA

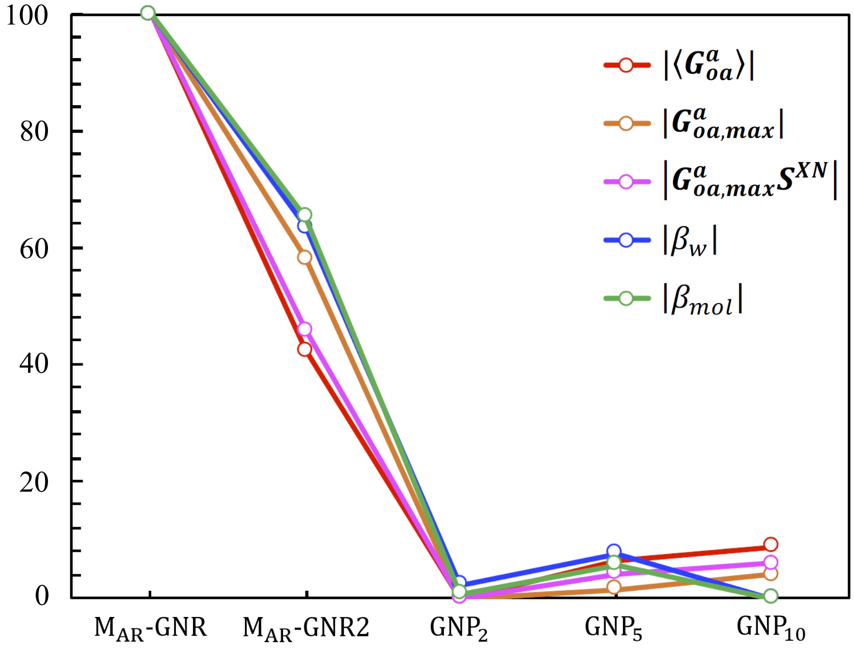

Fig. S17. Comparison between $|\langle G_{oa}^{a}\rangle|$, $|G_{oa,max}^{a}|$, and $\left|G_{oa,max}^{a}S^{XN}\right|$. Trends of previous $|\langle G_{oa}^{a}\rangle|$ data from reference [(*40*)] and newly calculated $|G_{oa,max}^{a}|$ as well as $\left|G_{oa,max}^{a}S^{XN}\right|$ in comparison to $|\beta_w|$ and $|\beta_{mol}|$; GNP_2 is a polyhedral quasi-spherical GNP with an average core diameter of d = 2 nm; M_{AR}-GNR2 is a gold nanorod with average dimensions of 51 × 23.5 nm (l × d) leading to a surface ligand-corrected aspect ratio of AR = 2.06.
